# Eco-friendly synthesis of silver nanoparticles as an unexplored application of photoredox catalysis

**DOI:** 10.1039/d6na00170j

**Published:** 2026-06-19

**Authors:** Willber D. Castro-Godoy, Luciana C. Schmidt, Juan E. Argüello, Adrián A. Heredia

**Affiliations:** a Laboratorio de Investigación en Productos Naturales (LIPN), Facultad de Química y Farmacia, Universidad de El Salvador Final Av. de Mártires y Héroes del 30 de Julio San Salvador 1101 El Salvador; b Instituto de Tecnología Química, Universitat Politècnica de València-Consejo Superior de Investigaciones Científicas Avda. de los Naranjos s/n, E-46022 Valencia Spain; c INFIQC-CONICET-UNC, Dpto. de Química Orgánica, Facultad de Ciencias Químicas, Universidad Nacional de Córdoba, Ciudad Universitaria X5000HUA Córdoba Argentina aheredia@unc.edu.ar juan.arguello@unc.edu.ar

## Abstract

A novel and sustainable methodology that merges photoredox catalysis and nanotechnology for the synthesis of AgNPs is reported. This methodology employs eosin Y as an organic photoredox catalyst under mild, aqueous, and visible-light irradiation conditions to rapidly obtain stabilized AgNPs. Optimal conditions were systematically determined, yielding AgNPs with average sizes of *ca.* 10–12 nm. A detailed reaction mechanism is also proposed, supported by experimental results and thermodynamic calculations. Furthermore, the synthesized AgNPs exhibited catalytic performance in the reduction of nitroarenes, demonstrating their potential synthetic application in organic chemistry.

## Abstract 2

The photocatalyzed synthesis of well-stabilized AgNPs employing eosin Y as an organic photocatalyst and visible light under sustainable reaction conditions is described.

## Graphical abstract

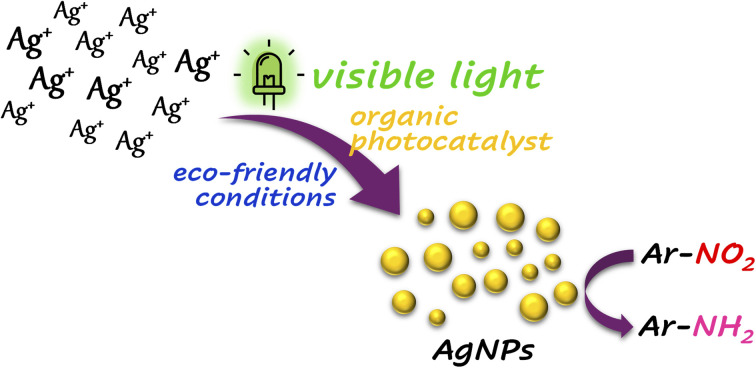

## Introduction

The synthesis of nanoscale metallic particles (less than 10 nm) remains a cornerstone of modern chemistry due to their unusual plasmonic properties, which allow their application in the fields of optics,^1–3^ microelectronics,^4,5^ catalysis,^6–11^ sensors,^12–14^ data storage,^15,16^ energy conversion,^17–20^ and even critical roles in biomedical challenges such as the COVID-19 pandemic,^21–27^ among others.^28–30^ It is well known that the size, structure, and physical, chemical and biological properties of metal nanoparticles are intrinsically ruled by the method of synthesis. Silver nanoparticles (AgNPs) can be obtained by chemical, physical, and biological methods, and given their current and growing interest, numerous reviews have summarized these methods.^31–38^ The chemical reduction of metal salts in the presence of a stabilizer is the most reported chemical approach.^35^ In the case of AgNPs, the most commonly used reducing agents are NaBH_4_, citrate and ascorbate salts through a bottom-up methodology. Photochemical methods use light to trigger the reduction reaction and have emerged as clean versatile alternatives that offer superior spatial and temporal resolution.^39^ Within this classification, direct photo-reduction involves solutions of metal cations irradiated with UV light, and the reduction is promoted by the protic solvents (water or alcohols) or their corresponding counterions;^40,41^ the photosensitized reduction relies on molecules that generate radical species under irradiation, which can transfer electrons to metal cations;^42^ finally, in photocatalytic depositions, semiconductors such as TiO_2_ are used to facilitate the transfer of electrons from the conduction band to the metal cations and to deposit the new nanoparticles on the semiconductor surface.^43^ Conversely, visible light-assisted photoredox catalysis is a tool that organic chemists have been intensively using over the last decade to enable chemical transformations ranging from simple reduction or oxidation reactions to challenging C–C and C-heteroatom new bond formation.^44–48^ Electronically excited photocatalysts – or their reduced or oxidized forms – facilitate electron transfer processes that lead to the desired chemical transformations. Substrates are often organic or organometallic molecules, but there are very few examples of electron transfer from excited organic dyes to inorganic metal precursors leading to nanomaterial formation.^49–53^ To date, molecular strategies have predominantly relied on synthetic organic sensitizers or specialized UV-laser systems; however, many of these approaches still present environmental drawbacks, such as the use of organic solvents or high-power energy-intensive irradiation sources.^54–57^ Concurrently, while bio-inspired protocols utilizing biological extracts and light have emerged as greener alternatives,^58^ they often suffer from poor kinetic control and mass-transport limitations. These biological matrices frequently lead to polydisperse particle sizes and extended reaction times, further complicated by unidentified reducing components that obscure the underlying reaction mechanisms. Consequently, developing a truly sustainable photocatalytic system that simultaneously achieves strict morphological control, rapid reaction kinetics, and mild operation conditions remains a highly desirable goal.

We have reported the reduction of Cu(ii) to Cu(i) for its application in the copper-catalyzed azide–alkyne cycloaddition reaction (CuAAC) using a visible light-assisted photoredox reaction.^59^ The reduction of Cu(ii) to Cu(i) was possible since the photogenerated reduced form of the dye, used as a photocatalyst, was able to transfer an electron to the metal cation (Δ*G* < 0).^60^ With this in mind, we hypothesize that by carefully modifying the reaction conditions, it would be possible to achieve a full reduction to the zero-oxidation state, thereby tuning the morphology, size, and physicochemical properties of the metal nanoparticles.

Herein, we present the use of eosin Y as an efficient organic photoredox catalyst for the photochemical reduction of silver ions in a completely aqueous medium, employing polyvinylpyrrolidone (PVP) as an innocuous stabilizer. In addition, the AgNPs obtained in this work are tested for the reduction of nitroarene substrates to highlight the practical advantages of this sustainable photoredox approach.

## Results and discussion

Our research began by studying the optimal reaction conditions for the synthesis of AgNPs, using their optical properties as an exceptional tool to monitor the reaction progress. AgNPs, like other metal nanoparticles, present unique plasmonic properties.^61^ Electromagnetic radiation can disturb electrons in the metallic nanoparticle: radiation promotes electronic oscillations around the whole nanoparticle, and as a consequence, radiation absorption occurs in certain regions of the electromagnetic spectrum. The resulting absorption spectrum depends exclusively on the nature of the metal, including shape, size, and even agglomerates or self-assembled structures.^62^ Since absorption spectra provide essential information about the nature of NPs, our study focuses on the analysis of UV-vis spectra of nanoparticles obtained under different reaction conditions. During the analysis, the position and absorbance of the plasmon band – around 400 nm for AgNPs – were analyzed, as well as the depression around 320 nm, which corresponds to the dielectric function imaginary component of the material.^63^ The full width at half maximum (FWHM) was also studied. The FWHM value of the corresponding plasmon resonance determines the dispersion of the NPs; a large FWHM corresponds to a broad peak and, consequently, polydispersity.^64–67^ This parameter is very sensitive to monodispersity; hence, narrow bands (low FWHM) are preferred when NPs are used as sensors, since small changes are rapidly detected during experiments.

The salt AgNO_3_ was used as the Ag(i) ion source, which is an easy-to-handle, readily available, and inexpensive metallic source; based on our previous results, eosin Y (EY) was considered the photocatalyst. Triethylamine (TEA) was used as the electron-donating sacrificial reagent. Finally, to ensure sustainable, greener conditions, water was chosen as the solvent for our study.

**Fig. 1:**
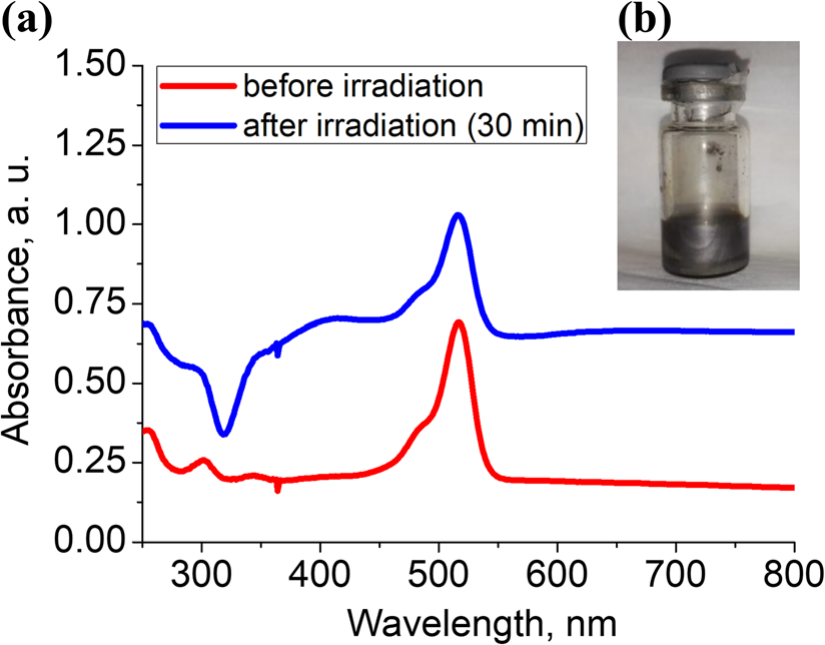
(a) UV-vis spectrum before (red line) and after (blue line) irradiation. (b) Photograph showing the appearance of the reaction after irradiation under initial conditions.

First, an aqueous solution of AgNO_3_ (25 mM, 0.05 mmol, 2 mL) was treated with 3 equiv. of TEA (0.15 mmol) and EY (1 mol%, 5 × 10^−4^ mmol). A brown precipitate was observed upon combining the silver salt and organic base in water, which was assigned to the oxide Ag_2_O.^68^ This precipitate was solubilized by continuous addition of TEA. The UV-vis spectrum of the mixture showed a band at 517 nm characteristic of EY and an increase in the baseline due to dispersion caused by the solid (Fig. 1a, red line). In all cases, the UV-vis spectra were acquired by taking 200 µL of the reaction mixture and transferring it to a quartz cell loaded with 3 mL of the solvent, which was eventually used as a blank. The mixture was purged with nitrogen for 15 minutes and irradiated with a 3 W green LED (522 nm) for 30 minutes. After irradiation, a metallic silver film was deposited on the vial, and a black suspended solid was observed (Fig. 1b). Greater scattering, the presence of EY, and a significant depression around 320 nm were observed in the UV-vis spectrum (Fig. 1a, blue line). Under these initial conditions, no AgNPs were obtained; however, the reduction reaction was evidenced by the formation of a silver film (Fig. 1b).

**Fig. 2:**
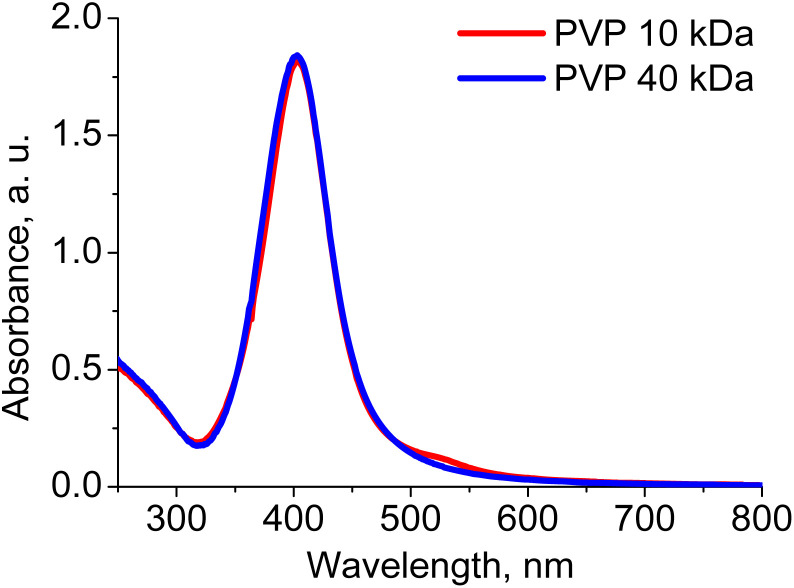
UV-vis spectra after irradiation employing PVP 10 kDa and PVP 40 kDa after 1 h irradiation of a mixture of AgNO_3_ (0.05 mmol, 25 mM), TEA (3 equiv.), and EY (1 mol%, 5 × 10^−4^ mmol) in 2 mL of an aqueous solution of PVP.

For the following experiments, the presence of stabilizing agents or ligands was considered. These are important components during the synthesis because they confine the growing material at a nanometric scale, stabilize the structure, control its shape, and prevent undesired agglomeration.^69^ First, mercaptosuccinic acid (MSA) was used as a ligand with a thiol group that can covalently bind to the surface of the NPs, and it also has carboxylic acid groups exposed on the surface, producing a nanomaterial dispersible in an aqueous medium. When the MSA concentration was 1% w/v, a red precipitate and an orange suspension were obtained after 30 minutes of irradiation. In this case, the UV-vis spectrum of the supernatant showed signals at wavelengths lower than 450 nm, indicating the absence of both NPs and photocatalyst (see Fig. S1(a) in the SI). The acidic pH of the medium can protonate the photocatalyst, deactivating its photocatalytic properties. When CTAB (cetyltrimethylammonium bromide) was used as a stabilizer, a greenish-brown mixture was obtained after irradiation. The UV-vis spectrum showed a broad absorption band with a maximum at 412 nm, corresponding to AgNPs, and a narrow band of unknown origin at 236 nm (see Fig. S1(b) in the SI). The resulting solution had a translucent light-yellow appearance, typical of AgNP suspensions. Even though AgNPs were obtained, their monodispersity was not optimal, as indicated by a high FWHM value (Table S1, entry 2). The water-soluble polymers PEG200, PVA, and PVP (40 kDa and 10 kDa) were also tested as stabilizers. When PEG was used, after irradiation, a black precipitate and a fluorescent-green supernatant were obtained. The UV-vis spectrum showed the absence of any plasmonic band, but a weak signal was observed at 490 nm, probably due to partial photodecomposition of the dye under these conditions (see Fig. S1(c) in the SI). A dark suspension was obtained when PVA was used as a stabilizer. The UV-vis spectrum showed a broad band around 390 nm and a depression at 320 nm, consistent with the presence of metallic nanoparticulate material (Table S1, entry 4). The decomposition of the photocatalyst was also observed as a very weak band at 490 nm. The UV-vis spectrum also showed significant scattering due to the suspended material (see Fig. S1(d) in the SI). Due to severe light scattering, reliable FWHM values could not be extracted for PVA-stabilized samples. Better results were found by using the non-toxic, inert, and biocompatible polymer PVP as a stabilizing agent. Even though PVP 10 kDa and 40 kDa were used, similar results were obtained. The reaction mixtures turned brown after irradiation, and their dilutions had a deep yellow color, typical of AgNP suspensions. The UV-vis spectra show narrow and well-defined absorption bands around 403 nm, with a depression at 320 nm characteristic of AgNPs. After 1 h of irradiation, the resulting spectrum of the reaction mixture with PVP 10 kDa showed a shoulder around 520 nm, probably due to the remaining EY (Fig. 2, red line), while in the case of PVP 40 kDa, this signal was absent, indicating the photobleaching of the photocatalyst (Fig. 2, blue line). Although the results were comparable in both cases, we continued this study with PVP 40 kDa, since EY would be removed from the reaction mixture after photocatalysis was achieved.

**Fig. 3:**
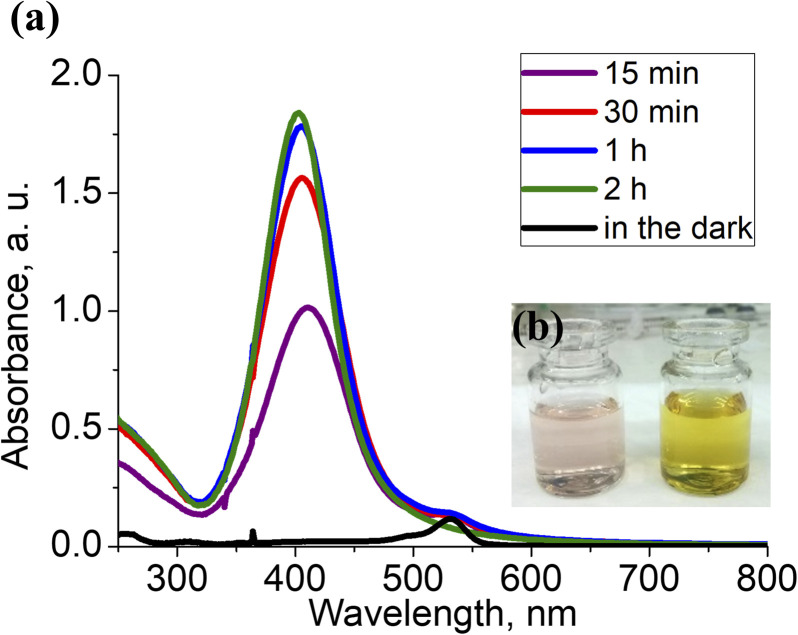
(a) UV-vis spectrum of the mixtures after irradiation for different reaction times. (b) Dilution (1 : 16) of the reaction mixtures performed in the dark (left) and under irradiated conditions (right).

Next, we studied the evolution of the surface plasmon resonance band with irradiation time (Table 1 and Fig. 3). The reagent mixture was then irradiated for 15 min, 30 min, 1 h, and 2 h under a nitrogen atmosphere. The presence of AgNPs was detected at each time point by observing a broad plasmonic resonance band around 410 nm, which shifted to 403 nm, while the absorbance increased to a value close to 2.0 after 1 h of irradiation. The system approaches monodispersity as irradiation time increases (FWHM decreases), indicating a controlled uniform growth. Due to the few differences found between the UV-vis spectra at 1 h and 2 h, we decided to consider 1 h as the optimal reaction time.

**Table 1:** Irradiation reaction time study^a^

Entry	Irradiation time	Plasmon resonance (UV-vis spectra)	
		*λ*_max_ (nm)	FWHM (nm)
1	15 min	410	89
2	30 min	405	87
3	1 h	405	77
4	2 h	403	68

a Reaction conditions: AgNO_3_ (0.05 mmol, 25 mM), TEA (3 equiv.), and EY (1 mol%, 5 × 10^−4^ mmol) in 2 mL of an aqueous solution of PVP 40 kDa (1% w/v), under a nitrogen atmosphere, irradiated with a 3 W green LED for the indicated time. Spectra were obtained from a 1 : 16 dilution.

**Fig. 4:**
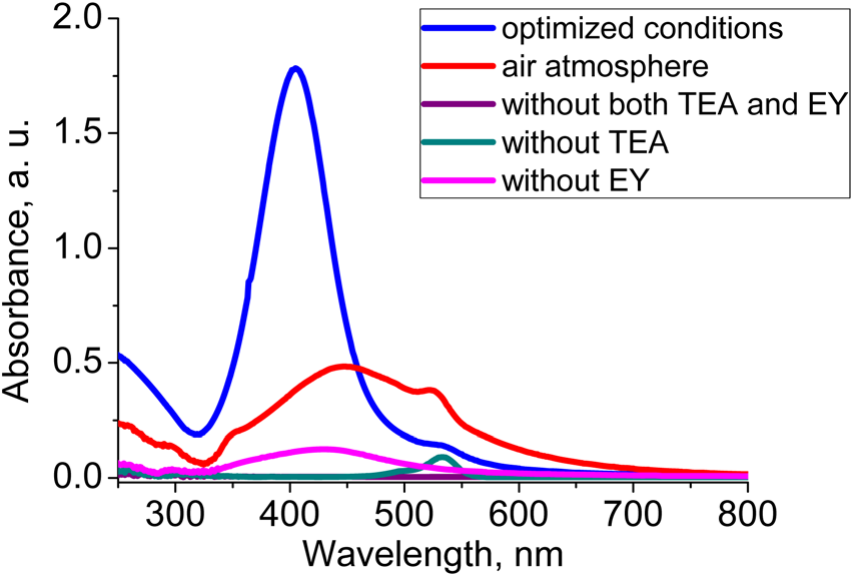
UV-vis spectra of reactions after irradiation under different control conditions.

Control experiments were performed, and the results are shown in Table 2 and Fig. 3b and 4. First, the reaction was carried out in the dark to ensure that it was assisted by visible light. In this case, the plasmon resonance of the AgNPs was absent, while the reaction mixture retained the initial color corresponding to EY (Fig. 3b).

**Table 2:** Control reactions^a^

Entry	Conditions	Plasmon resonance (UV-vis spectra)	
		*λ*_max_ (nm)	FWHM (nm)
1	None	405	78
2	Air atmosphere	450	279
3	Without TEA and EY	ND	
4	Without TEA	ND	
5	Without EY	430	163

a Reaction conditions: AgNO_3_ (0.05 mmol, 25 mM), TEA (3 equiv.), and EY (1 mol%, 5 × 10^−4^ mmol) in 2 mL of an aqueous solution of PVP 40 kDa (1% w/v), under a nitrogen atmosphere, irradiated with a 3 W green LED for 1 h. Spectra were obtained from a 1 : 16 dilution. ND: surface plasmon resonance not detected.

Control experiments are summarized in Table 2 and Fig. 4. The UV-vis spectrum of the reaction in air showed a broad plasmon resonance band with a maximum at 450 nm. This spectrum is characteristic of small, growing AgNPs, and oxygen from the air would slow down the growth of the first seeds (Table 2, entry 2). The formation of AgNPs was not evident in experiments where neither base nor EY was added, and even in the absence of base (in the presence of EY) (Table 2, entries 3 and 4). However, when EY was not added, very few AgNPs were obtained after 1 h of irradiation, indicating that their formation was induced by direct irradiation in the presence of TEA as a reducing agent (Table 2, entry 5). This last experiment proved that under the same reaction conditions, the presence of an organic dye as a photocatalyst significantly accelerated the process, while an inert atmosphere improved the quality of the synthesized nanoparticles.

Several organic dyes were also tested. The reaction conditions used are shown in Table 2, entry 1, except for the irradiation wavelength, which was selected based on the maximum absorption of each dye (Table 3 and Fig. S2 in the SI). As photocatalysts, EY, rhodamine 6G (R6G), fluorescein (FL), purpurin (PP), and quinizarin (QZ) were selected, while blue, green, and red LEDs were used as the irradiation sources. The best results were obtained for EY and FL under blue LED (467 nm) irradiation (Table 3, entries 1 and 2). For PP and QZ dyes, AgNP plasmon resonances were observed, but their intensities were low, with absorption maxima at 415 nm and 420 nm, respectively (Table 3, entries 3 and 4). The reaction with R6G showed a broad low-intensity plasmonic resonance band (Table 3, entry 4). EY, FL, R6G, and rose bengal (RB) were then tested under irradiation from a 3 W green LED (522 nm). Under these conditions, EY gives the best results (Table 3, entry 6). AgNPs from FL and RB showed a less intense and broader plasmon resonance, indicating the presence of small AgNPs (Table 3, entries 7 and 8). On the contrary, AgNP formation was not detected when R6G was irradiated at 467 and 522 nm, as no plasmon band was observed in the 380–450 nm region, despite strong dye absorption (Table 3, entries 5 and 9). Finally, MB was used under irradiation from a 3 W red LED (625 nm) without positive results (Table 3, entry 10). Although both EY and FL performed well under blue light conditions, we decided to use EY and green light as the optimal reaction conditions. Conversely, blue LED irradiation would excite the plasmon resonance of the formed AgNPs, favoring undesired reactions during synthesis; additionally, blue LED irradiation is more energetic and less selective.

**Table 3:** Study of dyes and LED sources^a^

Entry	Dye	LED	Plasmon resonance (UV-vis spectra)	
			*λ*_max_ (nm)	FWHM (nm)
1	EY	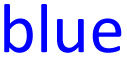	408	66
2	FL	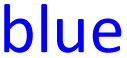	408	69
3	PP	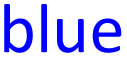	415	94
4	QZ	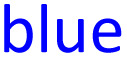	420	98
5	R6G	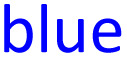	ND	
**6**	**EY**	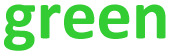	**404**	**73**
7	FL	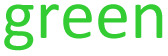	423	161
8	RB	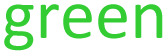	419	118
9	R6G	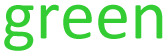	ND	
10	MB	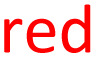	465	nd
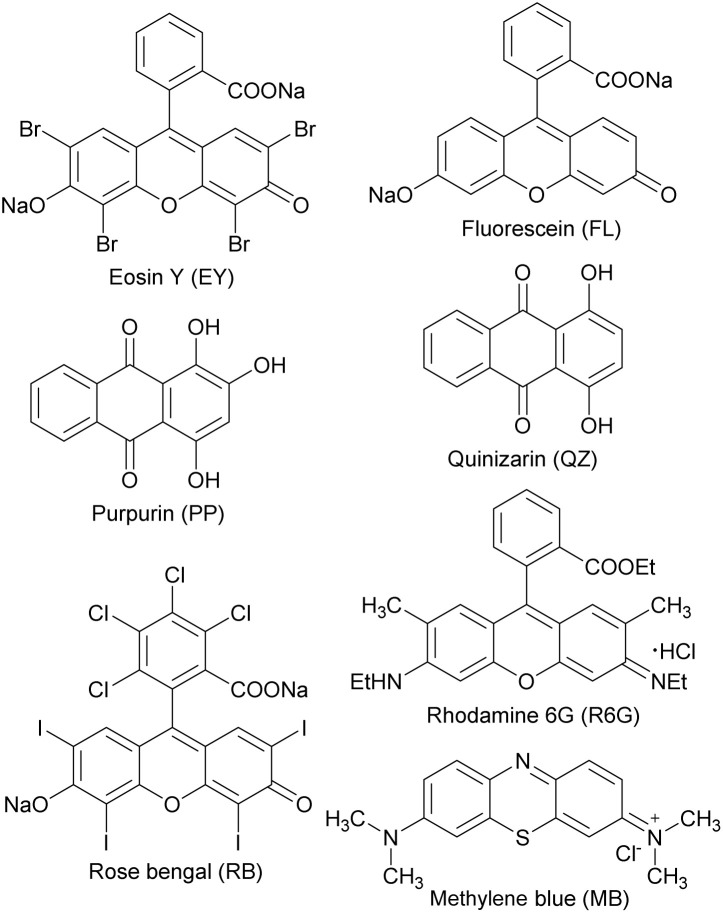				

a Reaction conditions: AgNO_3_ (0.05 mmol, 25 mM), TEA (3 equiv.), and dye (1 mol%, 5 × 10^−4^ mmol) in 2 mL of an aqueous solution containing PVP 40 kDa, under a nitrogen atmosphere, irradiated with the 3 W LED indicated in the table for 1 h. Spectra were obtained from a 1 : 16 dilution. ND: surface plasmon resonance not detected.

The optimal amount of photocatalyst was also tested. Therefore, different amounts of EY (0.1 mol%, 0.5 mol%, 1 mol%, 2 mol%, and 5 mol%) were employed (see Table S2 and Fig. S3 in the SI). A weak and broad plasmon resonance was observed with EY concentrations below 1%; for higher amounts of dye, a weaker plasmon resonance was detected, while a strong absorption signal from the photocatalyst was noticed. As a result, the optimal amount of photocatalyst was 1 mol% at an irradiation time of 1 h.

In addition, several electron-donating tertiary amines were also tested (Table S3 and Fig. S4). The best results were obtained using TEA and DIPEA (Table S3, entries 1 and 3). Less intense and broader plasmon resonances were acquired using TEOA and TMEDA (Table S3, entries 2 and 4), while no plasmon resonance was detected when EDTA was used as the reducing amine. As an optimal condition, TEA was chosen as our reducing agent due to its excellent efficiency and lower cost.

**Fig. 5:**
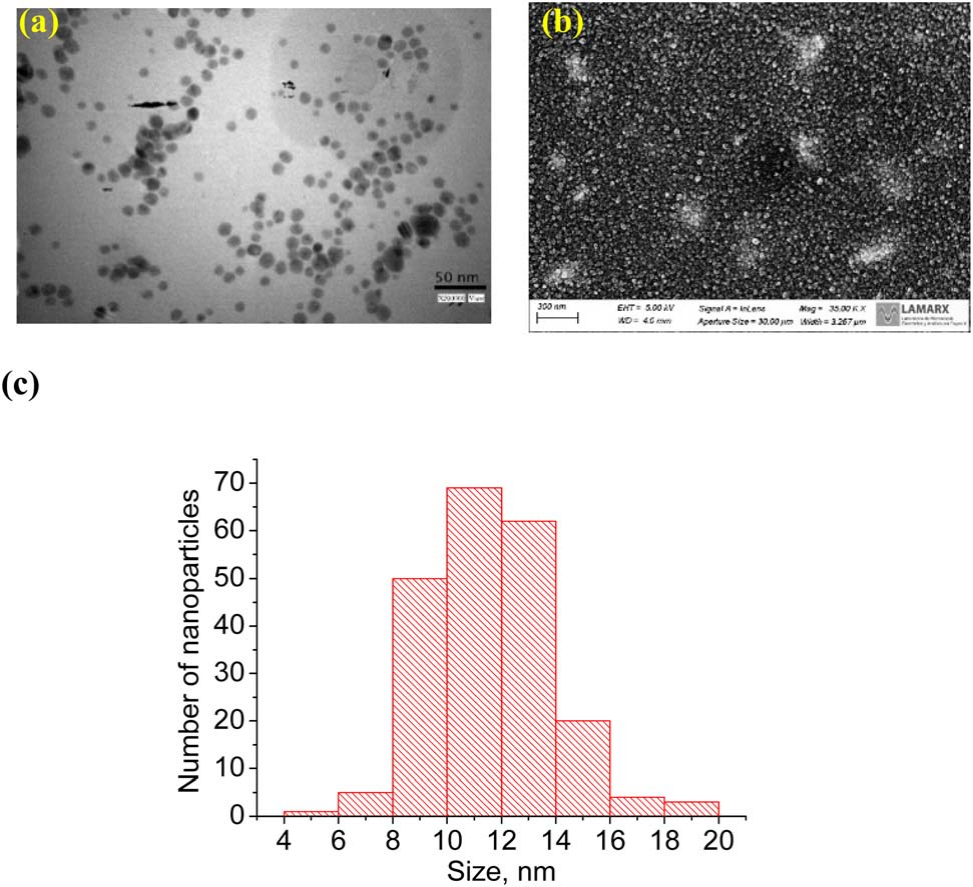
(a) TEM image of the synthetized AgNPs under the optimized conditions (scale bar = 50 nm). (b) SEM image of the synthesized AgNPs under the optimized conditions (scale bar = 300 nm). (c) Size distribution histogram of the photogenerated AgNPs under the optimized conditions.

Finally, a series of reactions using different amounts of 40 kDa PVP was performed. The results showed that well-defined and intense plasmon bands were observed in all cases (Table S4 and Fig. S5). However, when PVP 40 kDa at 2% w/v was employed, a narrow intense plasmon resonance band was observed; in addition, the total absence of the dye shoulder was also noticed. An identical result was obtained for 5% w/v of PVP 40 kDa. Our findings prove that as the amount of PVP increases, the stability of the AgNPs formed improves while the decomposition of the organic photocatalyst is accelerated.^70–72^ In summary, the optimal reaction conditions for the synthesis of AgNPs were AgNO_3_ (0.05 mmol), TEA (3 equiv.), and EY (1%) in 2 mL of PVP 40 kDa (2% w/v), under a nitrogen atmosphere and irradiated with a 3 W green LED for 1 h. The TEM and SEM images of the AgNPs obtained under the optimized reaction conditions showed spherical monodisperse nanoparticles with average sizes of approximately 10 nm to 12 nm (Fig. 5 and S7 in the SI).

**Fig. 6:**
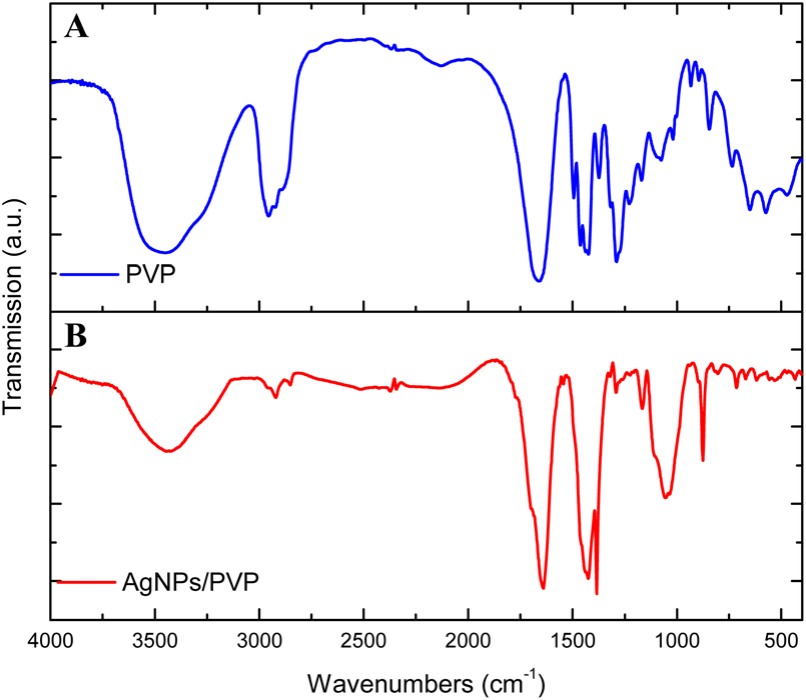
FTIR spectra of (A) PVP and (B) AgNPs/PVP.

The comparison between the FTIR spectrum of pure PVP and that of AgNPs/PVP provides strong spectroscopic evidence for the successful surface coordination of the PVP stabilizer onto the AgNPs (Fig. 6). The most significant difference lies in the redshift of the carbonyl (C=O) stretching vibration band, typically found around 1660 cm^−1^ in pure PVP (Fig. 6A); this band was observed at a lower wavenumber (1640 cm^−1^) in the AgNPs/PVP composite (Fig. 6B). Furthermore, other characteristic absorption bands observed in pure PVP were found at 1370 cm^−1^ and 1280 cm^−1^, corresponding to the vibration of the lactone structure and the stretching vibration of the C–N bond, respectively (Fig. 6A). The AgNPs/PVP spectrum shows changes in both the intensity and the shape of these bands (Fig. 6B). These spectral changes are attributed to the interaction between the oxygen atom of the PVP carbonyl group, acting as a capping agent, and the surface of the silver nanoparticles. This coordination accounts for the mechanism of AgNP stabilization.

### Reaction mechanism

**Fig. 7:**
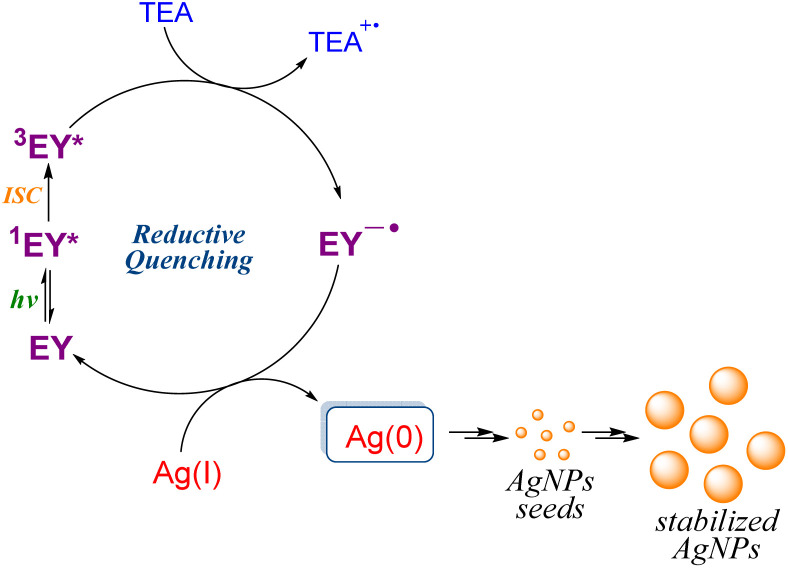
Proposed reaction mechanism.

The collected experimental results allow us to formulate a proposed reaction mechanism (Fig. 7). A photocatalyst, a tertiary amine, and visible light are essential for the photocatalyzed reduction of Ag(i) ions to Ag(0). Visible light generates electronically excited species of the photocatalyst. Thermodynamically, both singlet (^1^PC*) and triplet (^3^PC*) excited states could be involved in this transformation. Then, we can propose two plausible mechanisms starting from PC* (singlet (^1^PC*) and triplet (^3^PC*) excited states): first, reductive quenching, where PC* is reduced in the first step by the sacrificial reagent (Fig. 7); second, oxidative quenching, where PC* is oxidized concomitantly with the silver ion reduction (see the complete mechanism in the SI). Although both mechanisms are feasible, thermodynamic calculations support that only the former allows closure of a catalytic cycle, since the latter has a strongly endergonic process at the last step. The thermodynamic calculations of excited-state electron-transfer processes, as well as thermal processes, are indicated in the SI. Thus, by considering the reductive quenching, EY excited states (^1^EY* or ^3^EY*) could be reduced by TEA to generate the reduced species EY˙^−^ and the oxidized TEA˙^+^ (Δ*G* < 0). Nevertheless, our previous results showed that fluorescence quenching was not observed when a solution of EY was treated with TEA. Conversely, the consumption of ^3^EY* and concomitant formation of EY˙^−^ were evidenced by laser flash photolysis experiments, with a quenching rate constant of 7.6 × 10^6^ M^−1^ s^−1^.^60^ Fluorescence quenching experiments were performed by monitoring the emission of EY upon the addition of silver ions, both in the presence and absence of TEA. No appreciable quenching of the EY fluorescence was observed when Ag(i) was added in the absence of TEA, confirming that ^1^EY* does not participate in an oxidative quenching pathway (Fig. S6A). Conversely, quenching of the fluorescence was noticed when the addition of Ag(i) was performed in the presence of TEA (Fig. S6B). Nevertheless, this quenching is static in nature, attributed to the coordination of Ag(i) to the carboxylate group of EY, in agreement with previously reported literature data.^73^ These results rule out the contribution of the singlet excited state (^1^EY*) in the operative catalytic cycle, despite its thermodynamic feasibility. From a thermodynamic point of view, the reduced photocatalyst EY˙^−^ is reducing enough to transfer one electron to a Ag(i) ion, which is reduced to a free elemental silver atom. The Ag(0) atoms will form the first nucleation points (Ag seeds), and then stabilized AgNPs are obtained after successive reduction steps under a stabilizer-controlled growth. This last electron transfer step regenerates the photocatalyst in its fundamental initial state, restarting a new cycle.

### Comparative analysis of AgNP photocatalytic systems

To thoroughly contextualize the efficiency and novelty of our methodology, a comprehensive benchmarking against previously reported photochemical and photocatalytic systems for AgNP synthesis was conducted (see Table S5). A foundational example of the use of synthetic sensitizers was reported in 2005,^54^ where AgNPs (up to 20 nm) were synthesized using thionine in an organic mixture of ethanol/toluene under a high-power 250 W xenon lamp (Table S5, entry 1). More recent molecular approaches have utilized thioxanthone^55^ or benzophenone derivatives (Table S5, entries 2 and 3, respectively).^57^ However, these protocols still compromise environmental sustainability by relying on organic solvents such as acetonitrile, toluene or methanol, or requiring specialized laser diode equipment, while often leaving the catalytic applications of the resulting nanomaterial unexplored.

Alternatively, green synthesis protocols mediated by natural photoactive molecules or complex matrices have gained attention. For instance, a study proposed riboflavin as a metal-reduction promoter (Table S5, entry 4).^56^ Nevertheless, the absence of tailored sacrificial reagents and external stabilizing agents resulted in polydisperse and significantly larger AgNPs (57–73 nm) over a prolonged irradiation time. Similarly, bio-inspired methods utilizing plant,^74–76^ mushroom,^77^ or yeast extracts^78^ (Table S5, entries 5–8) under sunlight or white light irradiation face inherent challenges in kinetic control and particle uniformity, frequently requiring reaction times of 12–24 h or yielding large, poorly controlled colloids (up to 100 nm). Also, in these protocols, many components from the extract can act as reducing agents and stabilizers.^58,79^ The photoactive components are not properly identified, and the reaction mechanism is not adequately studied.

In contrast, our photoredox-driven system effectively bridges the gap between strict morphological control and environmental sustainability. By employing eosin Y as the photocatalyst in a completely aqueous medium, we successfully produced small, uniformly spherical AgNPs within a short reaction time and low-power green LED irradiation. Furthermore, while most reported photochemical protocols restrict their applications to antimicrobial assays or leave them unaddressed, the AgNPs obtained in this work demonstrate robust catalytic competence in the reduction of nitroarene, proving the practical synthetic utility and advantages of our sustainable approach.

### Applications in organic synthesis

The catalytic activity of the photocatalytically generated AgNPs was studied using the reduction reaction of nitroarenes (1) to anilines (2) in the presence of reducing agents. This reaction is important in both the laboratory and the chemical industry.^80^

First, 0.5 mmol of nitrobenzene 1a was placed in ethanol (2 mL) at 60 °C with 6 equiv. of a reducing agent (*e.g.*, hydrazine or NaBH_4_). Second, the AgNPs were added directly from the reaction crude without further treatment. These reactions were performed using 1 mg of Ag (9.26 × 10^−3^ mmol, 1.8 mol%), corresponding to 150 µL of reaction crude. In the presence of AgNPs, only NaBH_4_ reacted with 1a to generate products (2–4); thus, this reducing agent was selected for the optimization of reaction conditions (Table 4). Products 2a, 3a and 4a correspond to aniline, azobenzene and azoxybenzene, respectively.

**Table 4:** Reduction reactions of nitrobenzene (1a) using AgNPs^a^

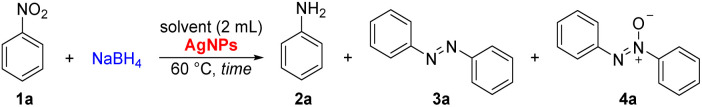						
Entry	Solvent	AgNPs (mol%)	Time (h)	2a (%)	3a (%)	4a (%)
1	EtOH	1.8	4	<1	68	10
2	EtOH	1.8	24	3	87	6
3	EtOH	3.6	4	90	3	7
**4**	**H**_**2**_ **O**	**1.8**	**4**	**86**	**6**	**7**
5	H_2_O	—	4	—	—	—

a Reaction conditions: 1a (0.5 mmol), NaBH_4_ (6 equiv.) and AgNPs (see mol% in table) in 2 mL of solvent, under an air atmosphere, stirred at 60 °C for the time shown in the table. Yields were determined by GC using the internal standard method.

When 1a was reduced in ethanol as the solvent, the main product was azobenzene 3a in 68% and 87% yields after 4 h and 24 h, respectively (Table 4, entries 1 and 2). The selectivity towards the amine 2a increases when the catalyst is added at 3.6 mol% in ethanol (Table 4, entry 3) or when water is used as the solvent (Table 4, entry 4). The latter condition resulted in a superior outcome since 2a was obtained with high selectivity and in very good yield. Furthermore, water was used as the solvent, and a low catalyst loading (without prior treatment) was employed. Finally, aniline was not observed in the absence of the catalyst (Table 4, entry 5).

**Scheme 1:**
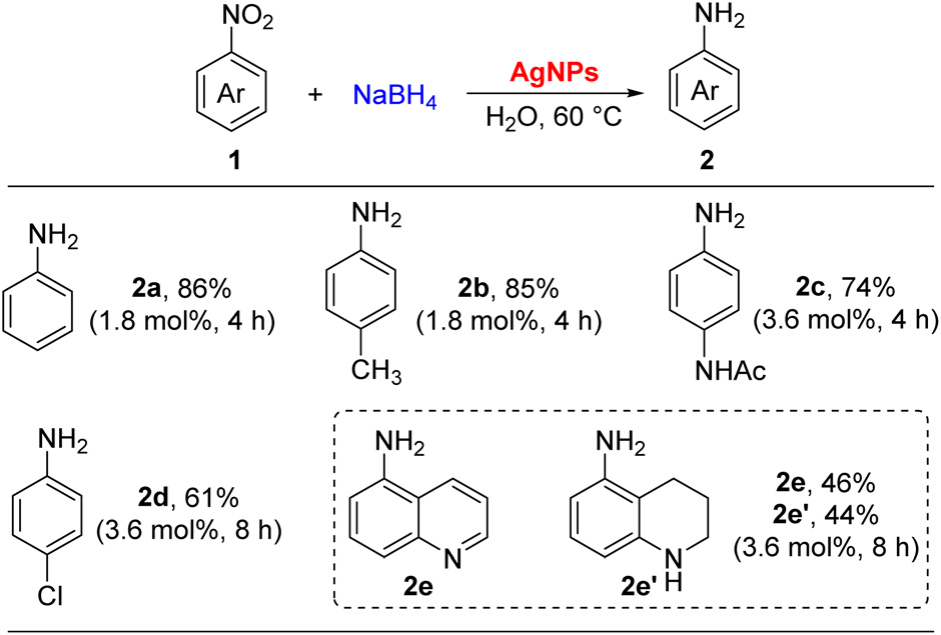
Reduction reactions of nitroarenes using AgNPs.

Other nitroarenes were used to explore the scope and limitations of this new methodology. Unfortunately, not all nitro compounds tested gave good yields under the same optimized conditions as 1a. The reaction scope obtained for compounds 1a–e is shown in Scheme 1. The reaction conditions optimized for each nitroarene are shown in parentheses (loading of AgNPs and reaction time), where a complete conversion of the substrate was observed.

Thus, *p*-toluidine (2b) was obtained in 85% yield using 4-nitrotoluene and 1.8 mol% of AgNPs after 4 h at 60 °C. On the other hand, 4-aminoacetanilide (2c) was produced in 74% yield when 4-nitroacetanilide was used, employing 3.6 mol% of AgNPs after 4 h. The nitroarene bearing a chlorine atom in the *para* position is less reactive, as 3.6 mol% of AgNPs and 8 h of reaction time were required to achieve complete conversion, yielding 61% of the corresponding chloroaniline 2d. Finally, when 5-nitroquinoline was used as the starting material, it was also necessary to increase the catalyst loading and reaction time to 3.8 mol% and 8 h, respectively. Two reduction products were observed: 5-aminoquinoline (2e) and 5-amino-1,2,3,4-tetrahydroquinoline (2e′) in 46% and 44% yields, respectively. This result suggests that the photogenerated nanocatalyst can promote not only nitro group reduction but also partial hydrogenation of heteroaromatic rings.

The results of this study demonstrate the catalytic activity of AgNPs produced *via* photocatalysis. The model reaction involving the catalytic reduction of nitro groups to the corresponding amines yields very promising results. The reaction mechanism for the reduction of nitro groups on metal nanoparticle surfaces is well established^81,82^ and is shown in Scheme S2. This methodology avoids the need to isolate and purify the nanocatalyst, and the studied reaction was found to be insensitive to the remaining dye or stabilizer. Furthermore, the presence of AgNPs was detected by UV-vis both before and after the reduction reaction (Fig. S6), demonstrating their high stability under strongly reducing conditions and moderate temperatures. These findings suggest that AgNPs are promising catalysts for other reactions of interest in organic synthetic chemistry. Furthermore, unlike most reported photochemical protocols where the synthetic utility of the resulting nanomaterial remains unexplored or limited to antimicrobial assays, the AgNPs obtained in this work demonstrate robust catalytic competence and excellent chemoselectivity in the reduction of nitroarene substrates, highlighting the practical advantages of this sustainable photoredox approach.

In conclusion, we were able to successfully carry out the photoreduction of Ag(i) ions using organic dyes and visible light, which led to the formation of well-stabilized AgNPs with excellent plasmonic and morphological properties. This represents one of the first examples of photoredox catalysis being employed for the synthesis of nanometric materials under mild and entirely sustainable conditions, using water as an environmentally benign and non-toxic solvent and PVP as an eco-compatible stabilizer. Furthermore, the catalytic activity of these AgNPs was demonstrated by studying the reduction reactions of nitroarenes and the hydrogenation of heteroaromatic rings.

## Experimental

### General methods

^1^H and ^13^C NMR spectra were recorded at 400.16 and 100.62 MHz, respectively, on a Bruker 400 spectrometer, and all spectra were reported in *δ* (ppm) relative to Me_4_Si, with CDCl_3_ as a solvent. Gas chromatographic analyses were performed on a chromatograph with a flame-ionization detector, using a 30 m capillary column with a 0.32 mm × 0.25 µm film thickness and a 5% phenylpolysiloxane phase. GC-MS analyses were performed on a spectrometer employing a 30 m × 0.25 mm x 0.25 µm column with a 5% phenylpolysiloxane phase. Ionization was achieved by electronic impact (70 eV), and detection was performed in positive mode.

### Chemicals

Ultrapure water was used without further purification. The reagents AgNO_3_, mercaptosuccinic acid (MSA), cetyltrimethylammonium bromide (CTAB), PEG200, PVA and PVP (10 kDa and 40 kDa) were all commercial samples used without further purification. The photocatalysts eosin Y disodium salt (EY), fluorescein (FL), rose bengal (RB), rhodamine 6G (R6G), purpurin (PP), quinizarin (QZ) and methylene blue (MB) were all high-purity commercial samples used without further purification. The tertiary amines triethylamine (TEA), triethanolamine (TEOA), *N*,*N*-diisopropylethylamine (DIPEA), *N*,*N*,*N*′,*N*′-tetramethylethylenediamine (TMEDA) and ethylenediaminetetraacetic acid (EDTA) were all high-purity commercial samples used without further purification.

### General experimental procedure for the synthesis of stabilized AgNPs (optimized condition)

The reaction was carried out in a 10 mL glass vial equipped with a rubber septum and a magnetic stirrer. AgNO_3_ (8.5 mg, 0.05 mmol), TEA (15.2 mg, 20.9 µL, 3 equiv.) and 100 µL of an aqueous solution of EY 5 mM (1 mol%) were dissolved in 2 mL of a PVP 40 kDa solution at 2% w/v. Nitrogen was bubbled through the mixture for 15 min. The reaction was irradiated with a 3 W green-LED (522 nm) and stirred under a nitrogen atmosphere for 1 h. Then, for characterization, a sample (200 µL) was dissolved in 3 mL of the stabilized solution, and the UV-vis spectrum was acquired. The full width at half maximum (FWHM) of UV-vis spectra was calculated by Lorentzian fitting (from 350 nm to 550 nm), where FWHM = *w* in 
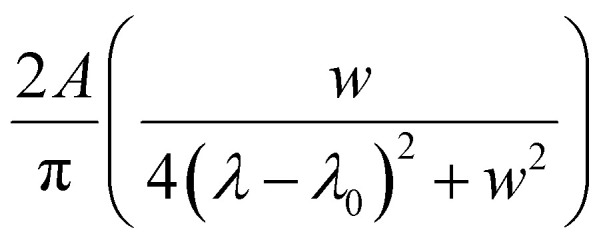
.

### General experimental procedures for the reduction of nitrobenzene (1a) using AgNPs

The reactions were carried out in a 10 mL Schlenk tube equipped with a magnetic stirrer. The tube was charged with water (2.0 mL). Nitroarene (1, 0.5 mmol), NaBH_4_ (6 equiv.) and 150 µL or 300 µL of crude AgNPs (corresponding to 1.8 or 3.6 mol%, respectively, see Scheme 1) were added and stirred at 60 °C for 4 or 8 h (see Scheme 1). The reaction mixture was cooled to room temperature. Diethyl acetate (15 mL) and water (15 mL) were added, and the mixture was stirred. The organic layer was separated, and the aqueous layer was extracted with diethyl ether (2 × 15 mL). The combined organic extract was dried over anhydrous Na_2_SO_4_, and the product was isolated by column chromatography (silica gel; eluent: hexane/ethyl acetate). The identities of all the products were confirmed by ^1^H, ^13^C and GCMS, and the spectroscopic data agreed with those previously reported in the literature.

### Spectroscopic data of synthesized anilines (2)

#### Aniline (2a)^83^

^1^H NMR (400 MHz, CDCl_3_): *δ* = 7.14 (td, *J* = 7.5, 3.7 Hz, 2H), 6.75 (td, *J* = 7.4, 1.0 Hz, 1H), 6.66 (dd, *J* = 7.5, 1.0 Hz, 2H), 3.60 (s, 2H). ^13^C NMR (100 MHz, CDCl_3_): *δ* = 146.5, 129.4, 118.6, 115.2. MS (EI): *m*/*z* (%) = 93 (100) [M]^+^, 66 (48).

#### *p*-Toluidine (2b)^84^

^1^H NMR (400 MHz, CDCl_3_): *δ* = 6.96 (d, *J* = 8.1 Hz, 2H), 6.61 (d, *J* = 8.1 Hz, 2H), 3.52 (s, 2H), 2.24 (s, 3H). ^13^C NMR (100 MHz, CDCl_3_): *δ* = 143.9, 129.9, 127.9, 115.4, 20.6. MS (EI): *m*/*z* (%) = 107 (68) [M]^+^, 106 (100), 79 (15).

#### 4-Aminoacetanilide (2c)^84^

^1^H NMR (400 MHz, acetone-d6): *δ* = 7.31 (d, *J* = 8.8 Hz, 2H), 6.59 (d, *J* = 8.8 Hz, 2H), 4.44 (s, 2H), 2.00 (s, 3H). ^13^C NMR (100 MHz, acetone-d6): *δ* = 168.0, 145.3, 130.5, 121.6, 115.1, 24.0. MS (EI): *m*/*z* (%) = 108 (100) [M]^+^, 107 (40), 81 (18), 80 (41).

#### 4-Chloroaniline (2d)^83^

^1^H NMR (400 MHz, CDCl_3_): *δ* = 7.09 (d, *J* = 8.6 Hz, 2H), 6.60 (d, *J* = 8.6 Hz, 2H), 3.64 (s, 2H). ^13^C NMR (100 MHz, CDCl_3_): *δ* = 145.1, 129.2, 123.3, 116.4. MS (EI): *m*/*z* (%) = 127 (100) [M]^+^, 100 (12), 92 (15).

#### 5-Aminoquinoline (2e)^85^

^1^H NMR (400 MHz, CDCl_3_): *δ* = 8.89 (dd, *J* = 4.1, 1.4 Hz, 1H), 8.18 (d, *J* = 8.5 Hz, 1H), 7.57 (d, *J* = 8.4 Hz, 1H), 7.51 (t, *J* = 7.5 Hz, 1H), 7.34 (dd, *J* = 8.5, 4.2 Hz, 1H), 6.82 (d, *J* = 7.3 Hz, 1H), 4.21 (s, 2H). ^13^C NMR (100 MHz, CDCl_3_): *δ* = 150.4, 149.3, 142.4, 130.1, 129.6, 120.3, 119.7, 118.9, 110.1. MS (EI): *m*/*z* (%) = 144 (100) [M]^+^, 117 (44), 90 (25).

#### 5-Amino-1,2,3,4-tetrahydroquinoline (2e′)^86^

^1^H NMR (400 MHz, CDCl_3_): *δ* = 6.81 (t, *J* = 7.9 Hz, 1H), 6.09 (dd, *J* = 7.8, 1.0 Hz, 1H), 6.01 (d, *J* = 7.9 Hz, 1H), 3.58 (s, 3H), 3.28–3.20 (m, 2H), 2.48 (t, *J* = 6.6 Hz, 2H), 2.05–1.97 (m, 2H). ^13^C NMR (100 MHz, CDCl_3_): *δ* = 145.8, 144.9, 127.0, 106.8, 105.6, 104.7, 41.4, 22.3, 21.5. MS (EI): *m*/*z* (%) = 148 (100) [M]^+^, 147 (75), 145 (14), 132 (27), 93 (20).

## Conclusions

We were able to successfully carry out the photoreduction of Ag(i) ions using organic dyes and visible light, which led to the formation of well-stabilized AgNPs with excellent plasmonic and morphological properties. This represents one of the first examples of photoredox catalysis being employed for the synthesis of nanometric materials under mild and entirely sustainable conditions, using water as an environmentally benign and non-toxic solvent and PVP as an eco-compatible stabilizer. Furthermore, the catalytic activity of these AgNPs was demonstrated by studying the reduction reactions of nitroarenes and the hydrogenation of heteroaromatic rings.

## Author contributions

Conceptualization: W. D. C.-G., A. A. H., L. C. S. and J. E. A. Formal analysis: W. D. C.-G., A. A. H., L. C. S. and J. E. A. Funding acquisition: A. A. H. and J. E. A. Investigation: W. D. C.-G., A. A. H. and J. E. A. Methodology: W. D. C.-G., A. A. H. and J. E. A. Project administration: A. A. H. and J. E. A. Resources: A. A. H. and J. E. A. Supervision: A. A. H. and J. E. A. Validation: A. A. H. and J. E. A. Visualization: W. D. C.-G., A. A. H. and J. E. A. Writing – original draft: A. A. H. and J. E. A. Writing – review and editing: W. D. C.-G., A. A. H., L. C. S. and J. E. A.

## Conflicts of interest

There are no conflicts to declare.

## Supplementary Material

NA-008-D6NA00170J-s001

## Data Availability

The data supporting this article have been included as part of the supplementary information (SI). Supplementary information: spectroscopic studies of stabilizers, amount of photocatalyst, sacrificial electron donors, thermodynamic studies and copies of ^1^H and ^13^C NMR spectra of synthesized anilines (2). See DOI: https://doi.org/10.1039/d6na00170j.
