# Supplementary material for: Eco-friendly synthesis of silver nanoparticles as an unexplored application of photoredox catalysis
Source: Nanoscale Adv. 2026 Jun 19;8(15):4292–301. doi: 10.1039/d6na00170j (PMC13324998; doi:10.1039/d6na00170j)
Supplement: NA-008-D6NA00170J-s001 [file NA-008-D6NA00170J-s001.pdf]

Supplementary Information (SI) for Nanoscale Advances.
This journal is © The Royal Society of Chemistry 2026

Supporting Information

Eco-Friendly Synthesis of Silver Nanoparticles as an Unexplored Application of Photoredox Catalysis

Willber D. Castro-Godoy, Luciana C. Schmidt, Juan E. Argüello* and Adrián A. Heredia*

Table of Contents

Table S1. Study of stabilizers S3
UV-vis spectra resulting from using different stabilizers S3
Table S2. Study of the amount of photocatalyst S4
Table S3. Study of tertiary amines as sacrificial electron donors S5
Table S4. Study of the concentration of the stabilizer S6
THERMODYNAMIC STUDIES S9
Table S5. Comparative analysis of AgNPs photocatalytic systems S12
^{1}H and ^{13}C NMR spectra of synthesized anilines (**2**) S13
REFERENCES S25

Table S1. Study of stabilizers

entry	stabilizer	plasmon resonance (UV-vis spectra)	
		λ_{max} (nm)	FWHM (nm)
**1**	MSA	ND	
**2**	CTAB	412	101
**3**	PEG200	ND	
**4**	PVA	392	nd
**5**	PVP10	404	66
**6**	PVP40	403	68

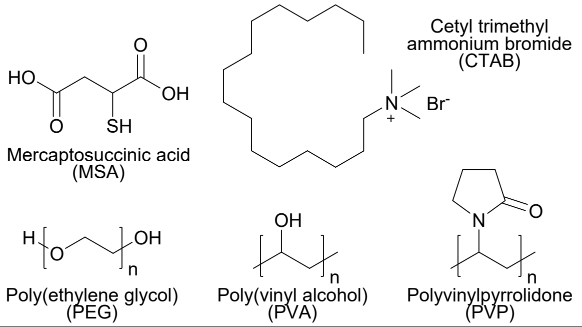

Reaction condition: $AgNO_3$ (0.05 mmol, 25 mM), TEA (3 equiv.), EY (1 mol%, $5x10^{-4}$ mmol) in 2 mL of an aqueous solution with the corresponding stabilizer at 1% w/v, under nitrogen atmosphere, irradiated with a 3W green LED for 30 minutes. Spectra obtained from a 1:16 dilution. ND: plasmon resonance not detected. nd: value not determined.

UV-vis spectra resulting from using different stabilizers

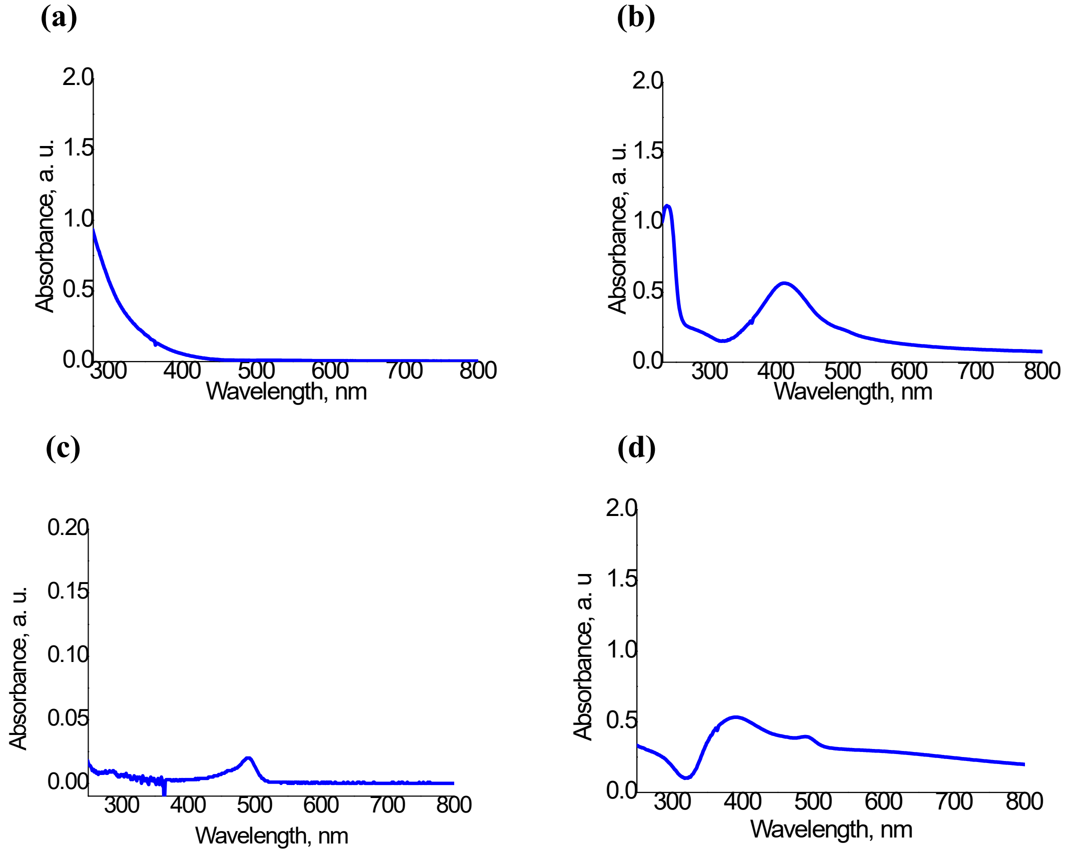

Figure S1. UV-vis spectra after irradiation employing (a) MSA (1% w/v); (b) CTAB (1% w/v); (c) PEG200 (1% w/v) and (d) PVA (1% w/v).

UV-vis spectra resulting from using different organic dyes and irradiation sources

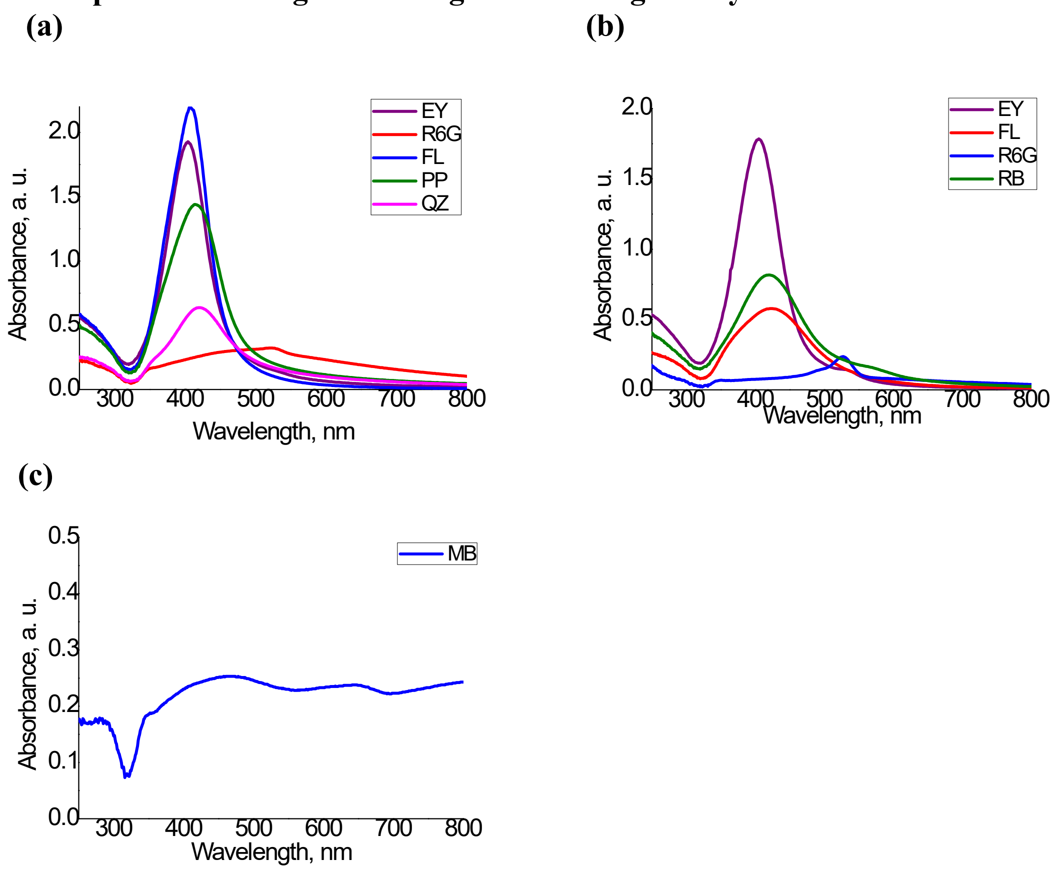

Figure S2. UV-vis spectra after irradiation with (a) a 3 W blue-LED (467 nm); (b) a 3 W green-LED (522 nm) and (c) a 3 W red-LED (625 nm).

Table S2. Study of the amount of photocatalyst

entry	EY (mol%)	plasmon resonance (UV-vis spectra)	
		λ_{max} (nm)	FWHM (nm)
**1**	0.1	413	110
**2**	0.5	406	95
**3**	1	404	74
**4**	2	401	81
**5**	5	398	77

Reaction condition: $AgNO_3$ (0.05 mmol, 25 mM), TEA (3 equiv.), EY (indicated in the table) in 2 mL of an aqueous solution with PVP 40kDa (1% w/v), under nitrogen atmosphere, irradiated with a 3W green LED for 1 h. Spectra obtained from a 1:16 dilution.

UV-vis spectra resulting from using different amounts of EY

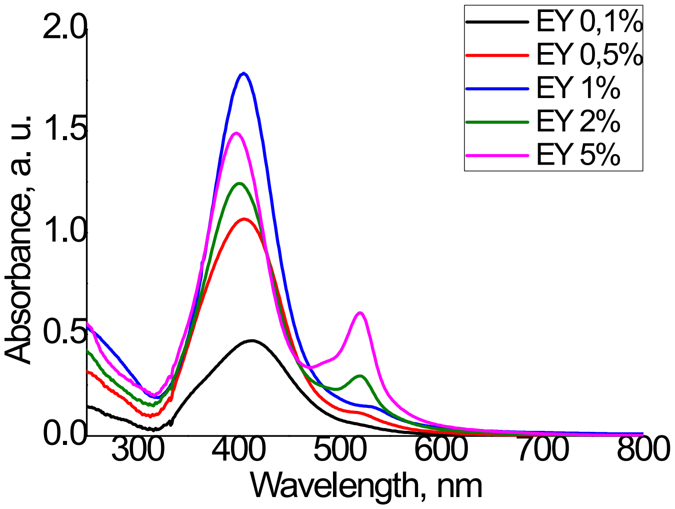

Figure S3. UV-vis spectra of the reaction mixture after irradiation using different amounts of EY.

Table S3. Study of tertiary amines as sacrificial electron donors

entry	amine	plasmon resonance (UV-vis spectra)	
		λ_{max} (nm)	FWHM (nm)
**1**	TEA	405	79
**2**	TEOA	403	86
**3**	DIPEA	407	83
**4**	TMEDA	415	103
**5**	EDTA	ND	

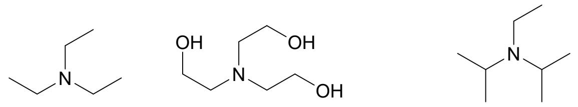

Triethylamine (TEA) Triethanolamine (TEA) *N*,*N*-diisopropylethylamine (DIPEA)

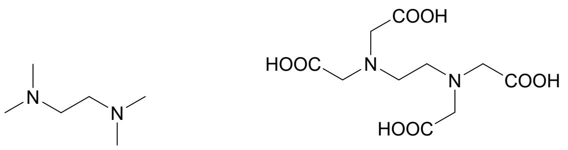

Tetramethylethylenediamine (TMEDA) Ethylenediaminetetraacetic acid (EDTA)

Reaction condition: $AgNO_3$ (0.05 mmol, 25 mM), amine (3 equiv.), EY (1 mol%, 5 x 10^{-4} mmol) in 2 mL of an aqueous solution with PVP 40 kDa (1% w/v), under nitrogen atmosphere, irradiated with a 3W green LED for 1 h. Spectra obtained from a 1:16 dilution. ND: plasmon resonance not detected.

UV-vis spectra resulting from different tertiary amines as sacrificial electron donors

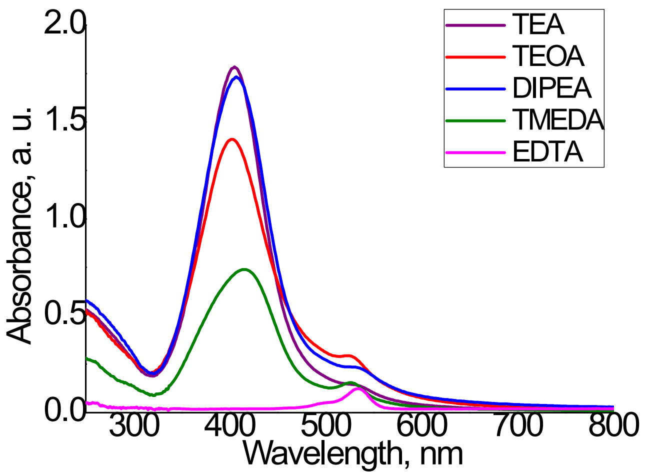

Figure S4. UV-vis spectra of the reaction mixture after irradiation using different tertiary amines as sacrificial electron donors.

Table S4. Study of the concentration of the stabilizer

entry	PVP (%w/v)	plasmon resonance (UV-vis spectra)	
		λ_{max} (nm)	FWHM (nm)
**1**	0.1	410	95
**2**	0.5	407	77
**3**	1	405	76
**4**	2	405	71
**5**	5	407	78

Reaction condition: $AgNO_3$ (0.05 mmol, 25 mM), TEA (3 equiv.), EY (1 mol%) in 2 mL of an aqueous solution with 40 kDa PVP (concentration indicated in table), under nitrogen atmosphere, irradiated with a 3 W green LED for 1 h. Spectra obtained from a 1:16 dilution.

UV-vis spectra resulting from using different amounts of PVP 40 kDa

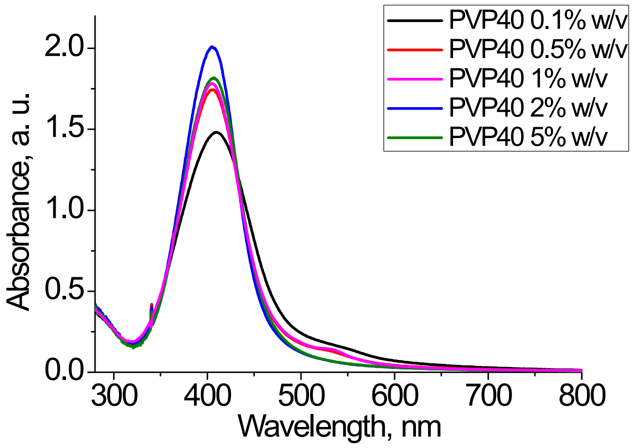

Figure S5. (a) UV-vis spectra of the reaction mixture after irradiation using different amounts of PVP 40 kDa.

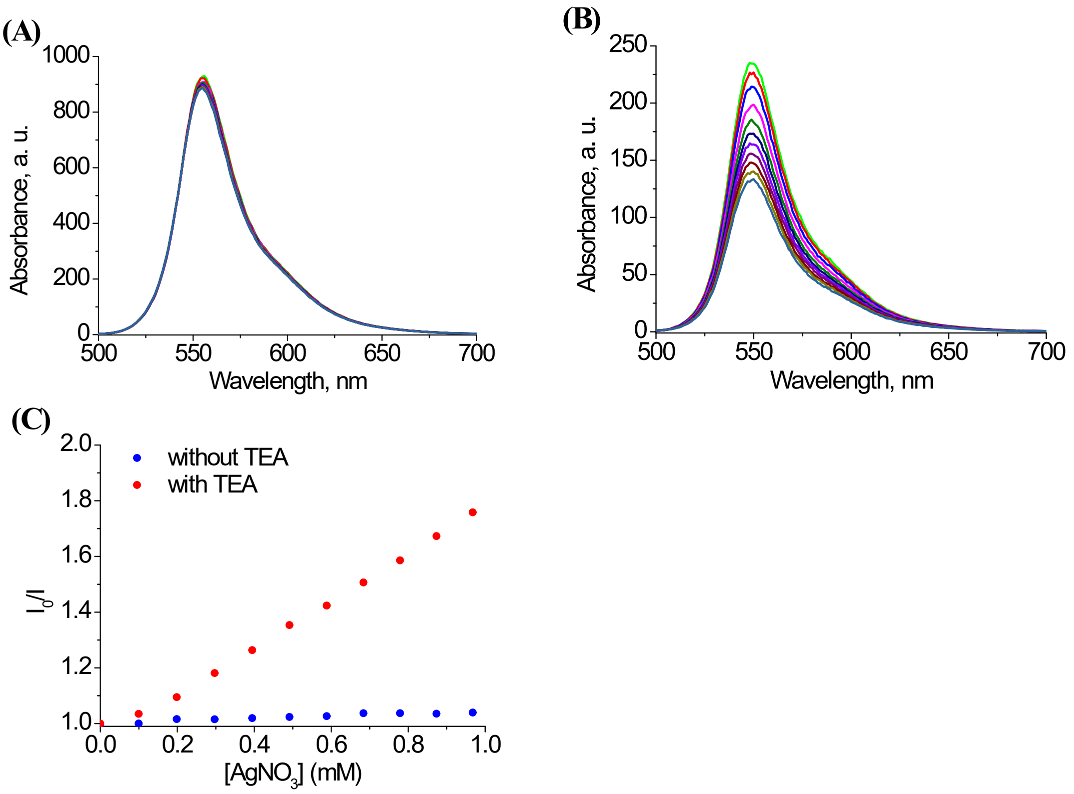

Figure S6. Fluorescence quenching experiments. (A) Steady-state fluorescence (λ_{exc} = 460 nm) of EY (1 x 10^{-2} mM) in PVP 2% w/v upon addition of $AgNO_3$ (up to 1 mM). (B) Steady-state fluorescence (λ_{exc} = 460 nm) of EY (1 x 10^{-2} mM) in PVP 2% w/v in the presence of TEA (3 mM) upon addition of $AgNO_3$ (up to 1 mM). (C) Fluorescence quenching Stern-Volmer plot (K_{SV}^{woTEA}= 0.047 mM^{-1}; K_{SV}^{wTEA}=0.810 mM^{-1}).

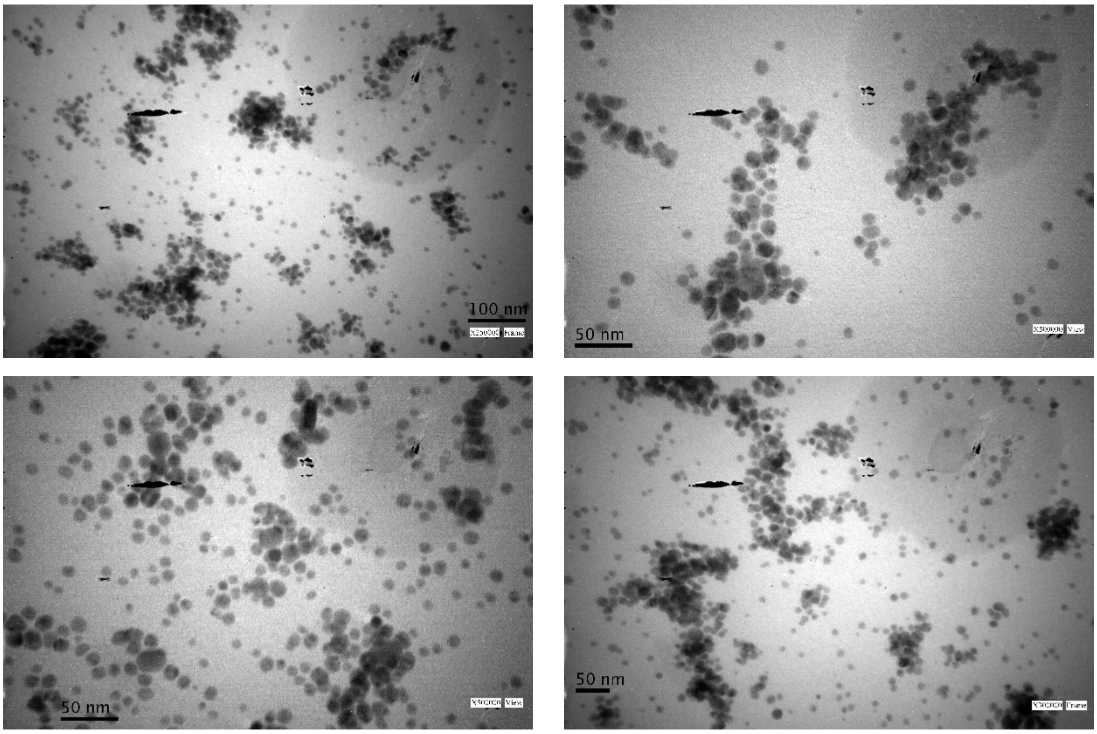

Figure S7. TEM images of the synthetized AgNPs under the optimized conditions.

IRRADIATION SETUP

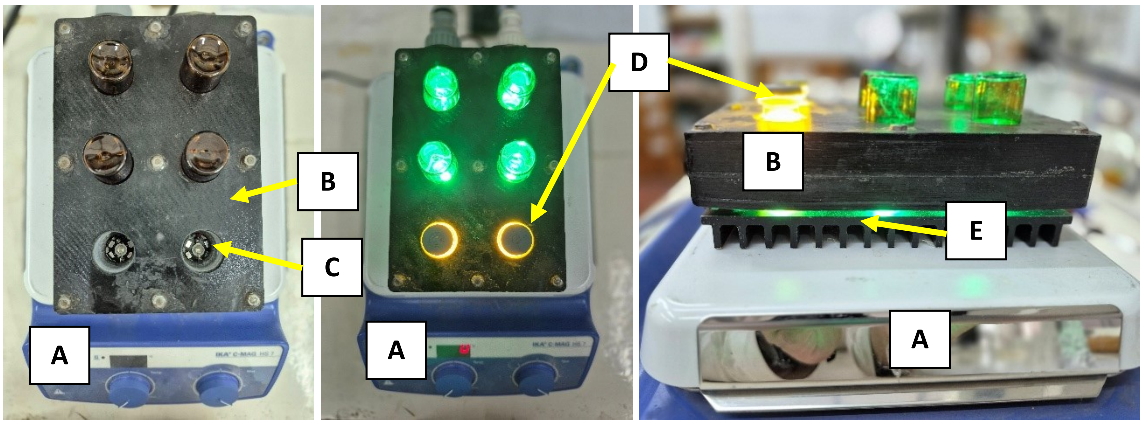

Figure S8. Homemade photoreactor: **A**: Magnetic stirrer. **B**: Cooling rack connected to a temperature controlled recirculatory (25 °C). **C**: 3W green LED (522 nm). **D**: Glass reaction vial. **E**: Aluminum heatsink supporting six 3W green LED lights on it.

THERMODYNAMIC STUDIES

Possible mechanistic scenarios are shown in Scheme S1. To assess the exergonicity of each step and cycle, thermodynamic calculations were developed.

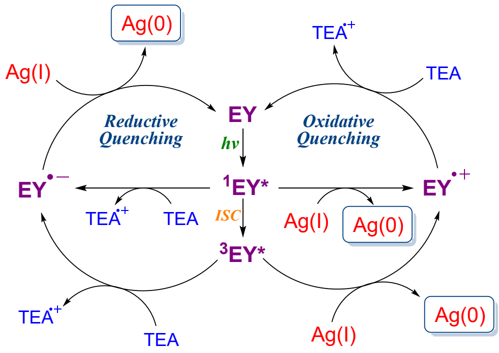

Scheme S1. Reduction photocatalyzed of Ag(I) to Ag(0). Both reductive and oxidative quenching are showed.

Gibbs free energy equation for photoinduced processes (Rehm-Weller equation):[1]

$$\Delta G = -[E(A) - E(D)] - E_{excited\ state} + \Delta E_{coul}$$

Gibbs free energy equation for thermal redox processes:

$$\Delta G = - n F [E(A) - E(D)]$$

where n = 1 mol e^- in all cases and $F = 96485$ J V^{-1} mol^{-1}

Redox potential:

E (EY / $EY^{\bullet-}$) = – 1.06 V vs SCE[2]
E ($EY^{\bullet+}$ / EY) = + 0.72 V vs SCE[2]
E (Ag(I) / Ag(0)) = + 0.552 V vs SCE[3]
E ($TEA^{\bullet+}$/ TEA) = + 0.96 V vs SCE[4]

Excited state energies

E_S (EY) = + 2.31 eV[5]

E_T (EY) = + 1.91 eV[5]

Coulombic term

is relatively small for polar solvents, ca. 0.06 eV in CH_3CN, and is therefore neglected.

All potentials are expressed in volts and energies converted to eV where appropriate.

Reductive quenching

- Oxidation of TEA from the singlet EY

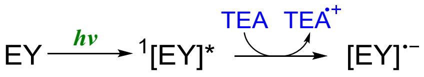

$\Delta G = -[-1.06 - 0.96] - 2.31\ \text{eV} = -0.29\ \text{eV} =$ **$-$ 6.69 kcal mol^{-1}**

- Oxidation of TEA from the triplet EY

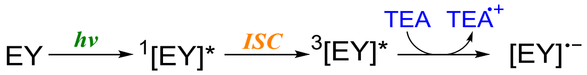

$\Delta G = -[-1.06 - 0.96] - 1.91\ \text{eV} = +0.11\ \text{eV} =$ **+ 2.54 kcal mol^{-1}**

- Reduction of Ag(I) from the EY$^{\cdot -}$

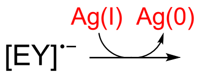

$\Delta G = -\ n\ F\ (0.552\ \text{V} - (-1.06\ \text{V})) =$ **$-$ 155.53 kJ mol^{-1} = $-$ 37.17 kcal mol^{-1}**

Oxidative quenching

- Reduction of Ag(I) from singlet EY

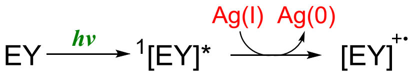

$\Delta G = -[0.552\ \text{V} - 0.72\ \text{V}] - 2.31\ \text{eV} = -2.142\ \text{eV} =$ **$-$ 49.39 kcal mol^{-1}**

- Reduction of Ag(I) from triplet EY

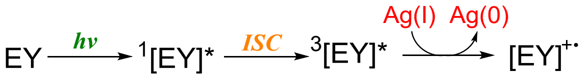

$\Delta G = -[0.552\ \text{V} - 0.72\ \text{V}] - 1.91\ \text{eV} = -1.742\ \text{eV} =$ **$-$ 40.17 kcal mol^{-1}**

- Oxidation of TEA from EY$^{\cdot +}$

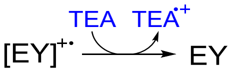

$\Delta G = -\ n\ F\ (0.72\ \text{V} - 0.96\ \text{V}) =$ **+ 23.16 kJ mol^{-1} = + 5.53 kcal mol^{-1}**

MECHANISM OF REDUCTION OF NITROARENES CATALIZED BY AgNPs[6]

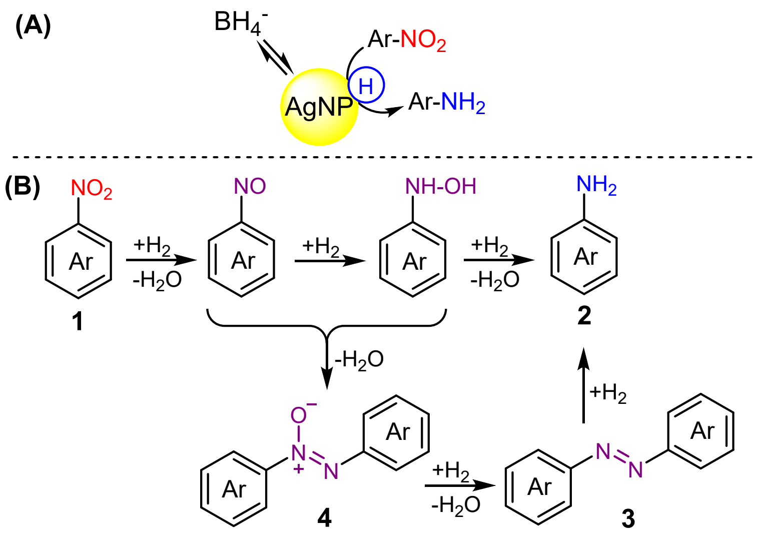

Scheme S2. (A) Catalytic activation of H_2 generated from BH_4^- ions and adsorption and reduction of nitroarenes on the AgNP surface. (B) Proposed mechanism for the catalytic reduction of nitroarenes with activated hydrogen by AgNPs.

UV-vis spectra of photogenerated AgNPs before and after the reduction of nitrobenzene

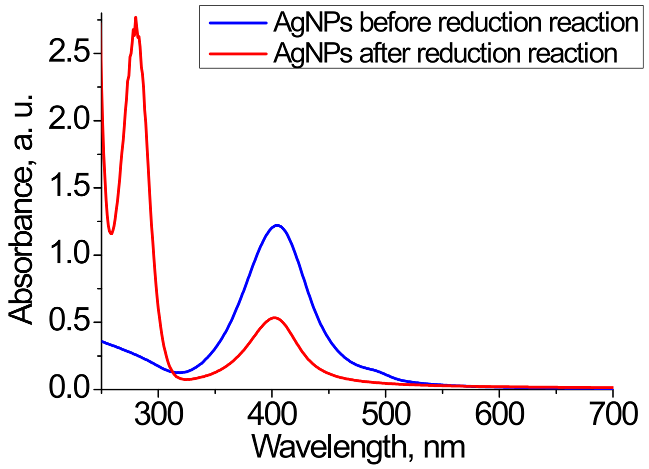

Figure S9. UV-vis spectra of AgNPs before and after the catalytic reduction of nitrobenzene.

Table S5. Comparative analysis of AgNPs photocatalytic systems

Entry	photoactive molecule	light source (λ_{em})	reaction time	solvent	stabilizing/capping agent	shape (size)	application	reference
**1**	thionine	250 W xenon lamp (530 nm cutoff filter)	1.5 h	Ethanol/toluene	thionine	Spherical (up to 20 nm)	-	*Chem. Mater.* **2005,** *17,* 5404-5410
**2**	thioxantones	laser diode (377 nm)	15 min	acetonitrile	thioxantone	Spherical (4.8 nm)	-	*J. Phys. Chem. C* **2010,** *114,* 10396–10402
**3**	benzophenone derivatives	35 W blue LED (419 nm)	25 seg	methanol	benzophenone derivatives	Spherical (up to 24 nm)	-	*ACS Omega* **2023**, *8*, 3207−3220
**4**	riboflavin	HPLN 125 W (405-435 nm)	6 h	water	riboflavin	spherical and polydisperse (57-73 nm)	antimicrobial activities	*J. Mol. Struct.* **2023**, *1289*, 135863
**5**	*Matricaria chamomilla* L.herb extract	sunlight	5 min	water	plant extract	Spherical (about 26 nm)	dye degradation	*Biomolecules* **2020**, *10*, 1604
**6**	*Withania somnifera* leaf powder	sunlight	12 h	water	biomolecules from the pant	Pseudo-spherical (5-30 nm)	antimicrobial activities	*J. Photochem. Photobiol. B: Biol.* **2014**, *132*, 45-55
**7**	flavoproteins from *Pleurotus citrinopileatus* extract	sunlight	3 h	water	biomolecules from the mushroom	Spherical (7 nm)	antibacterial activities	*J. Photochem. Photobiol. B: Biol.* **2018**, *188*, 42-49
**8**	*Saccharomyces cerevisiae* extract	White light	24 h	water	biomolecules from extract	Spherical (100 nm)	-	*Bioprocess Biosyst. Eng.* **2024**, 7, 1669–1682
This work	***eosin Y***	***3 W green LED (522 nm)***	***1 h***	***water***	***PVP 40 (2% w/v)***	***Spherical (10-12 nm)***	***reduction of nitroarenes***	**-**

^{1}H and ^{13}C NMR spectra of synthesized anilines (2)
^{1}H NMR. Aniline (2a)

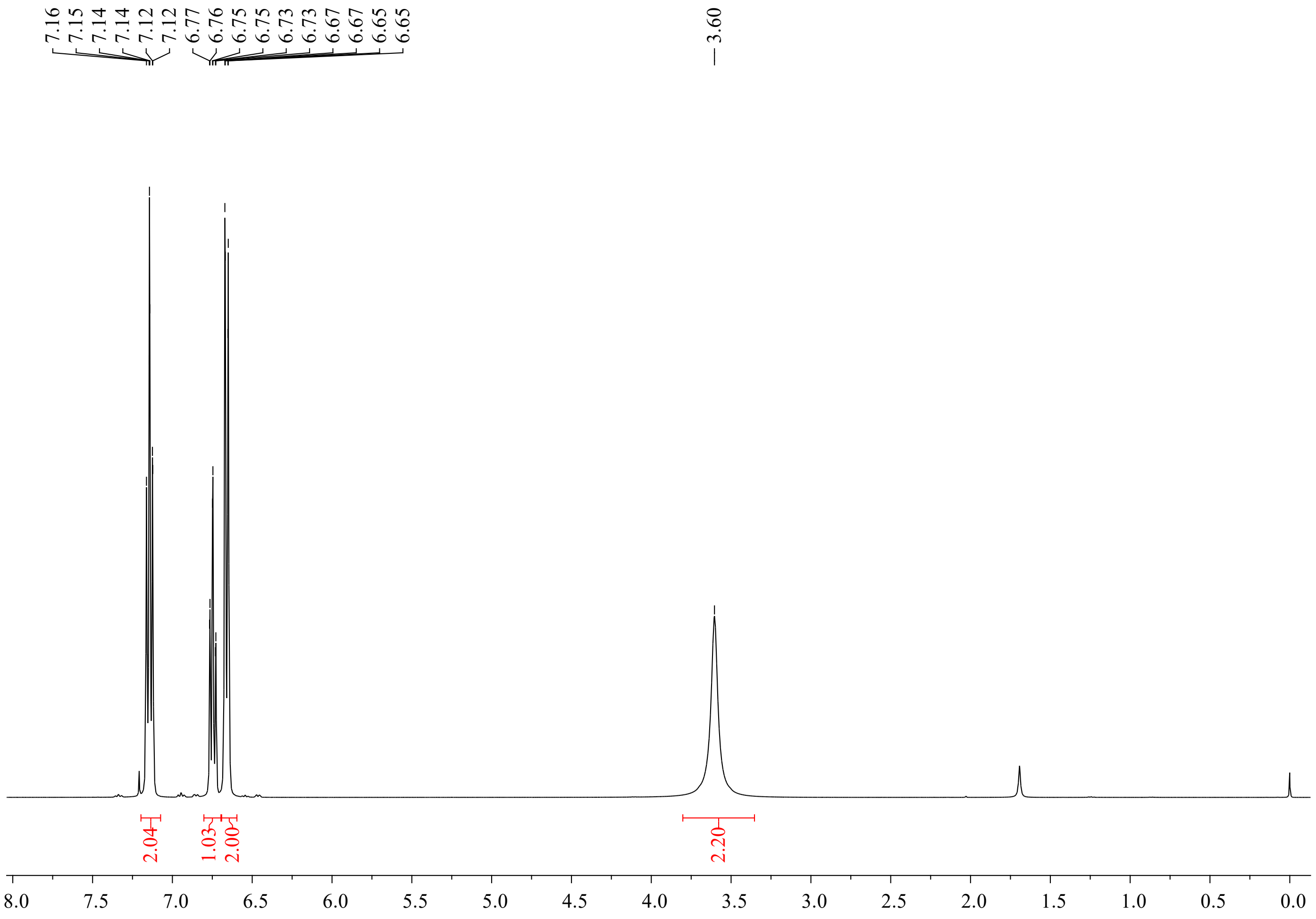

^{13}C NMR. Aniline (2a)

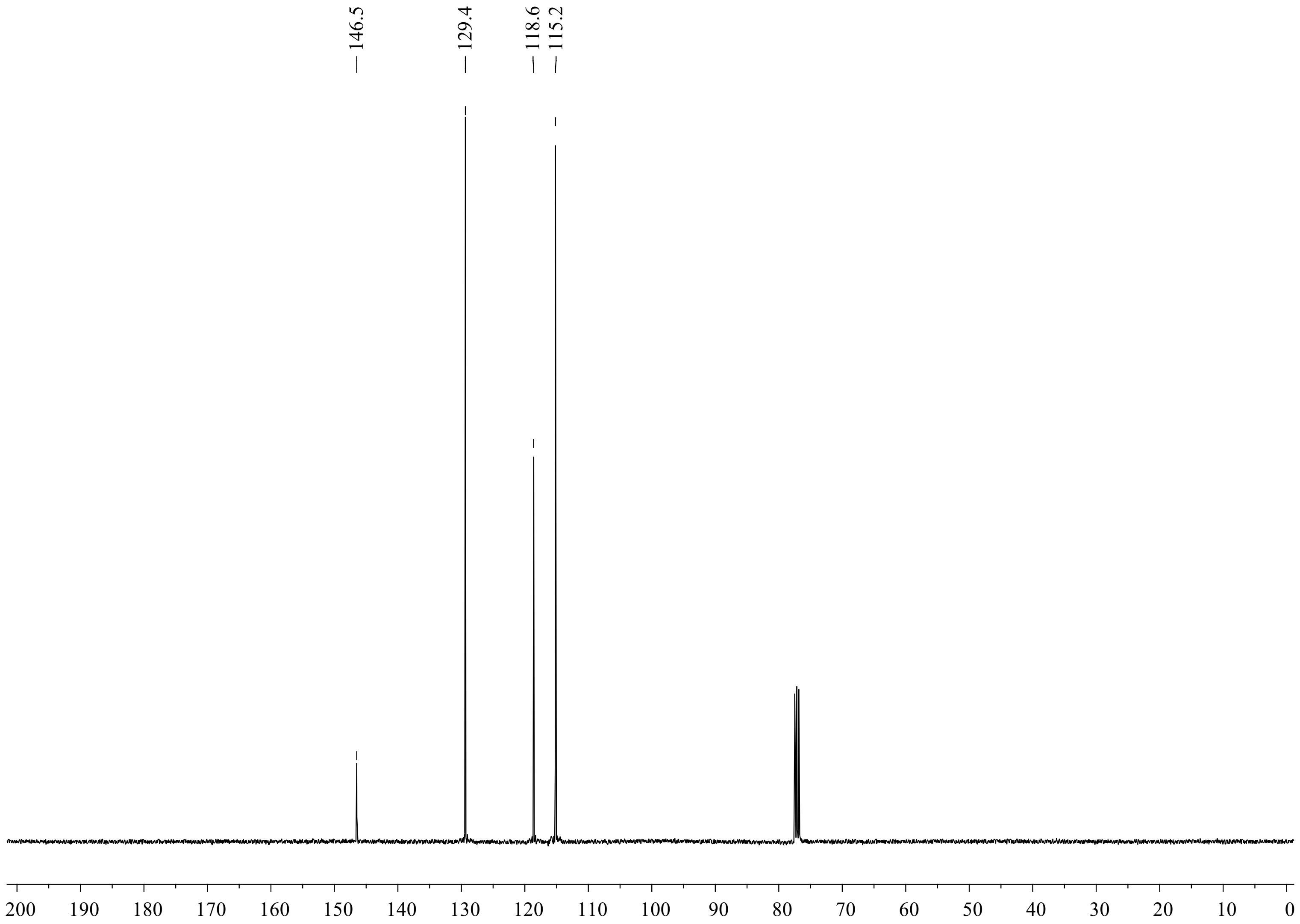

^{1}H NMR. *p*-toluidine (2b)

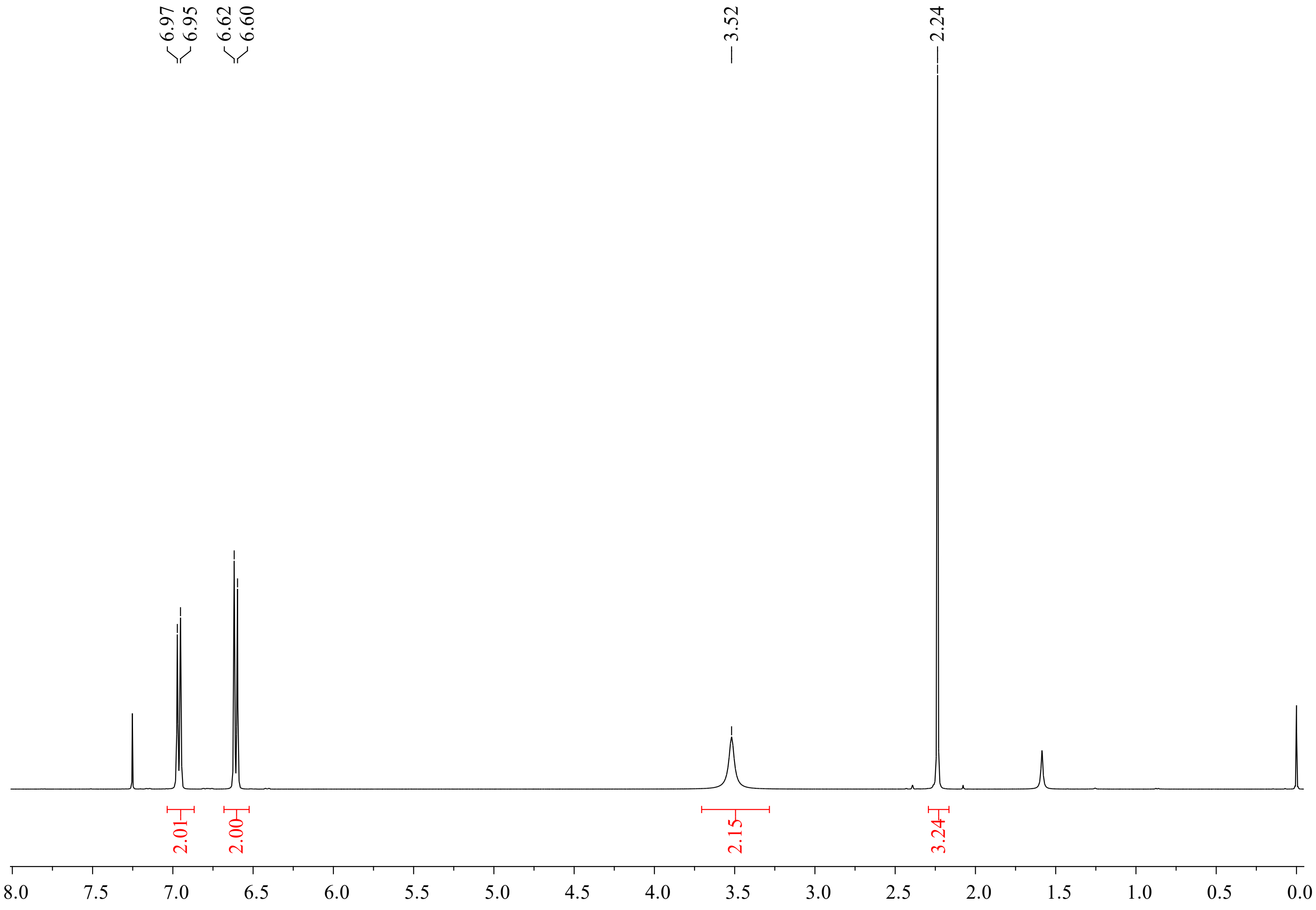

^{13}C NMR. *p*-toluidine (2b)

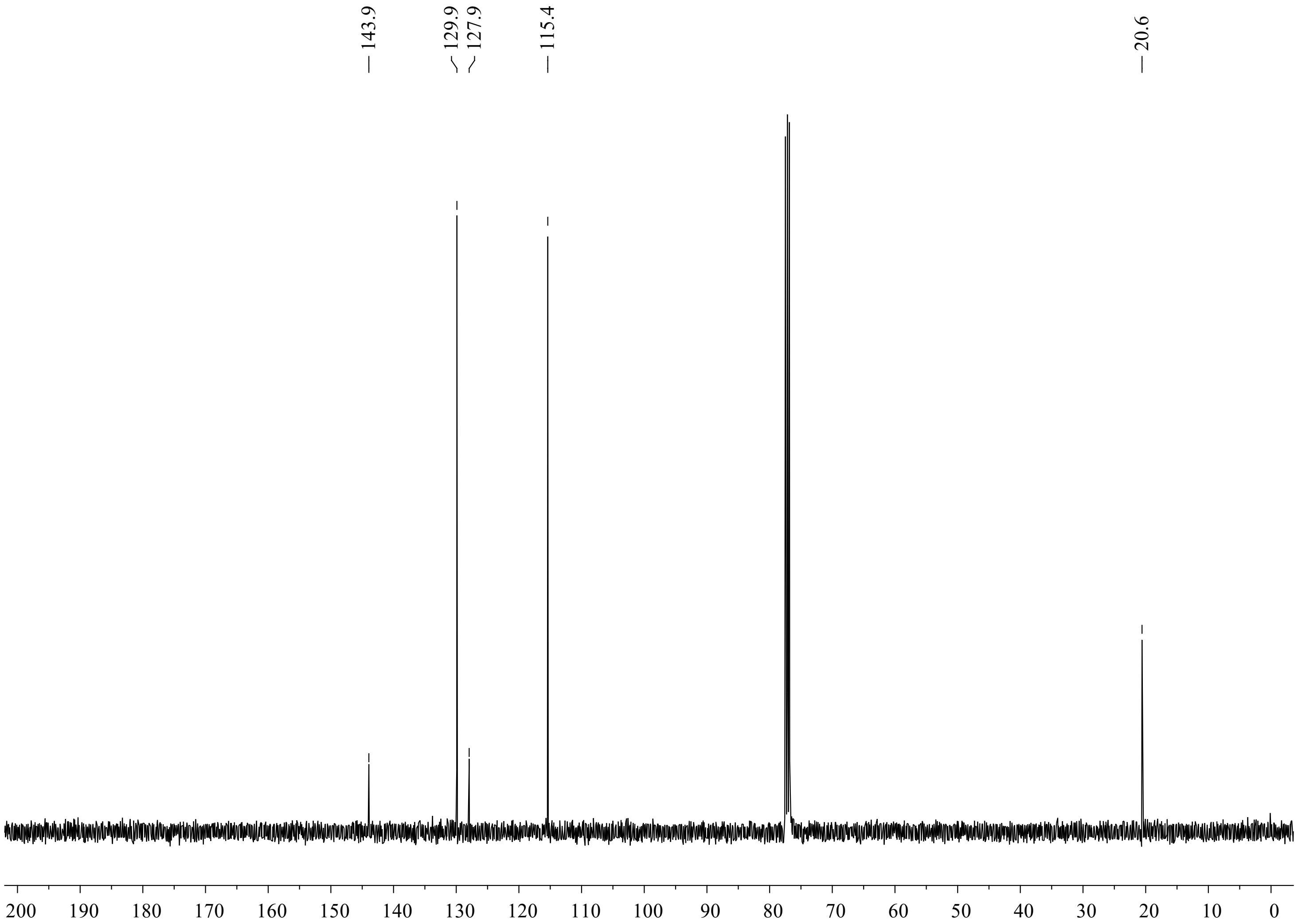

^{1}H NMR. 4-aminoacetanilide (2c)

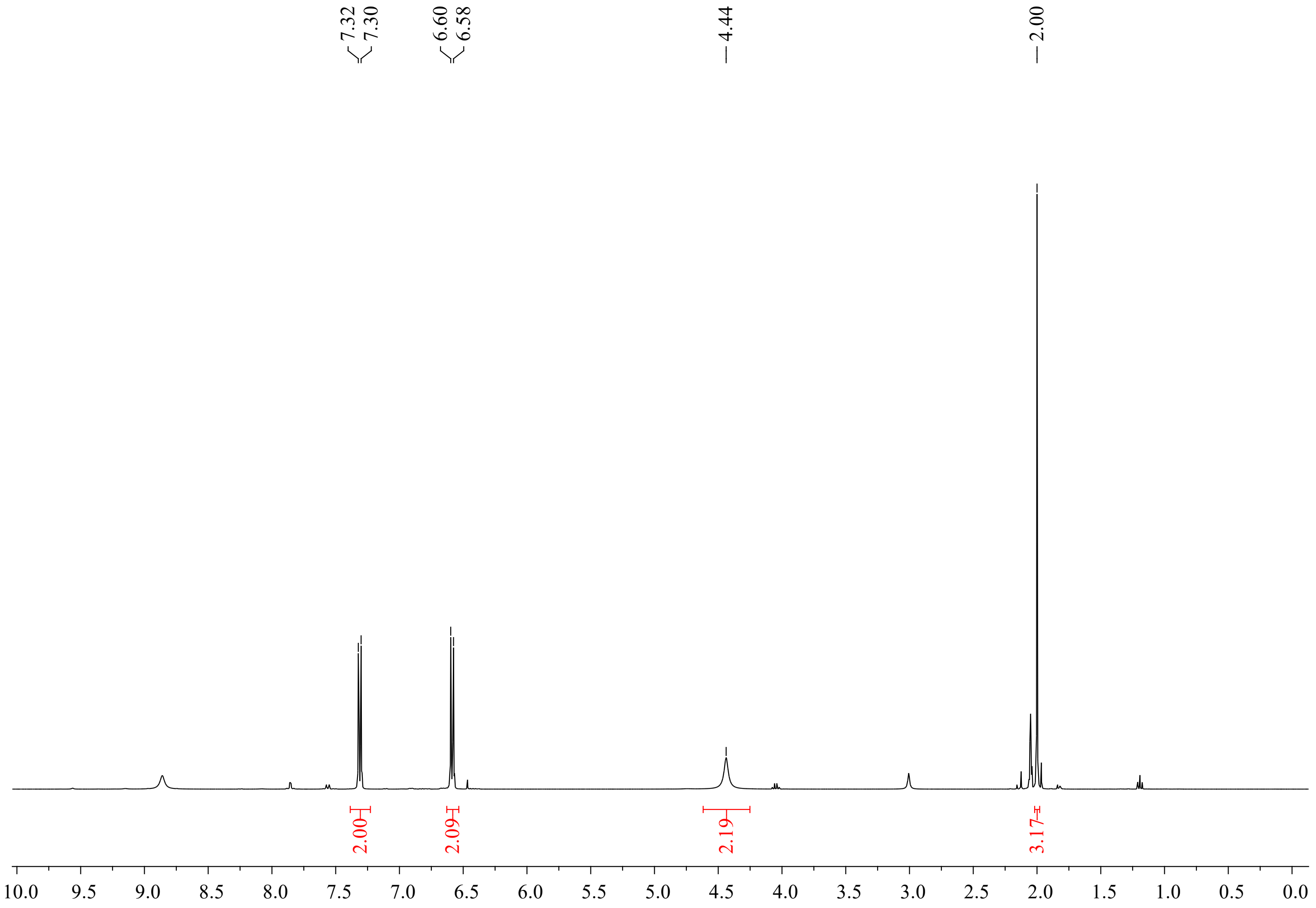

^{13}C NMR. 4-aminoacetanilide (2c)

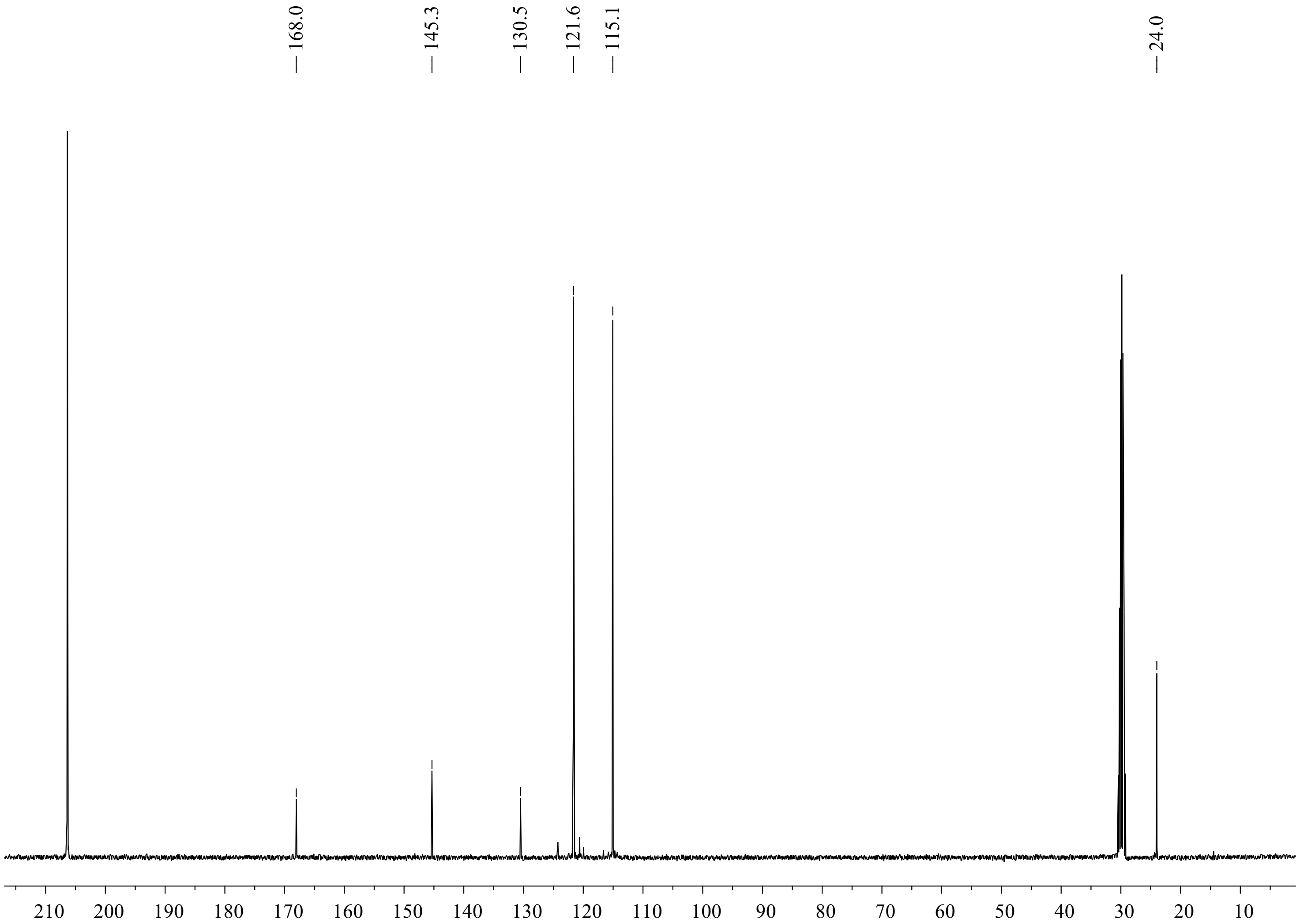

^{1}H NMR. 4-chloroaniline (2d)

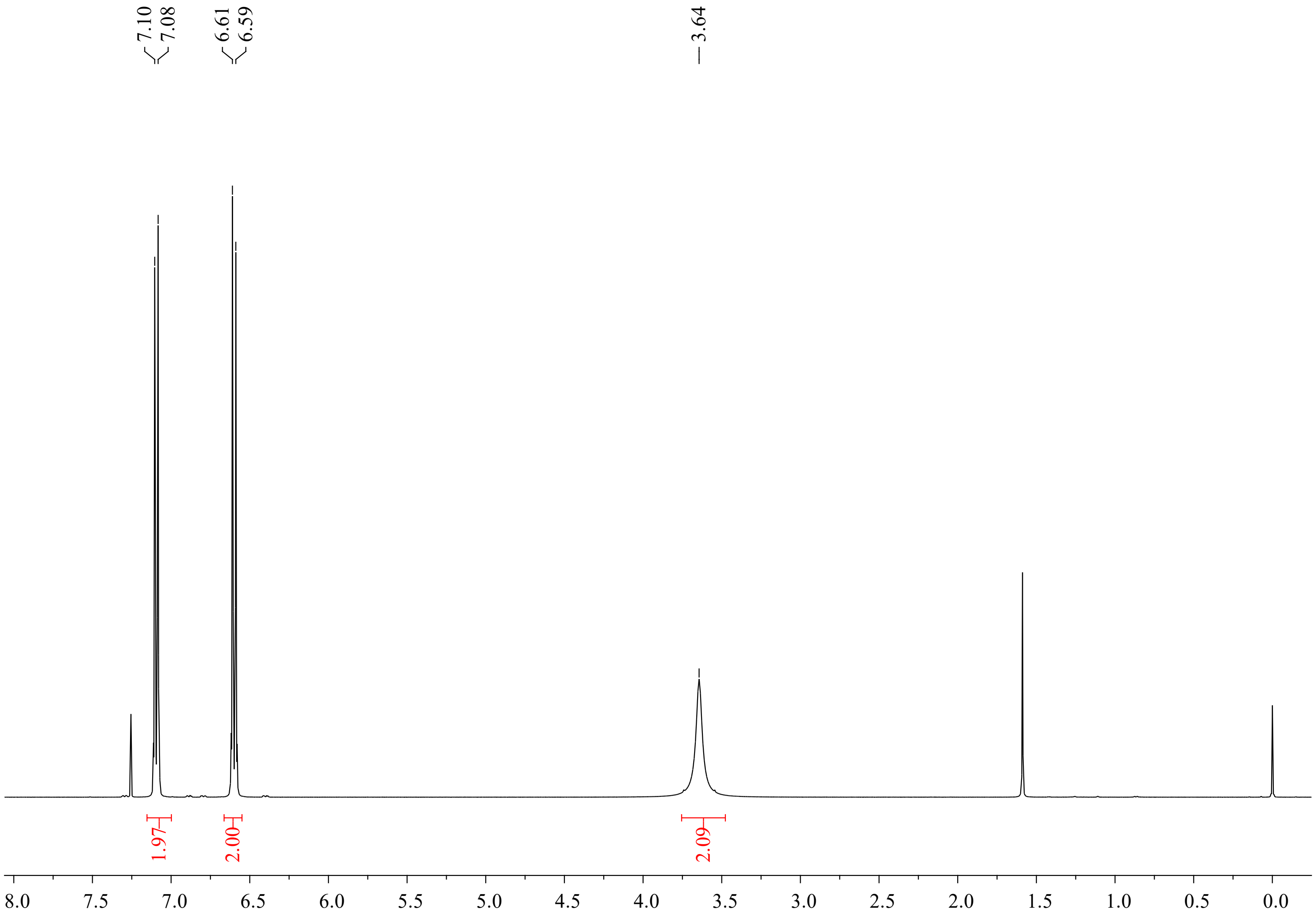

^{13}C NMR. 4-chloroaniline (2d)

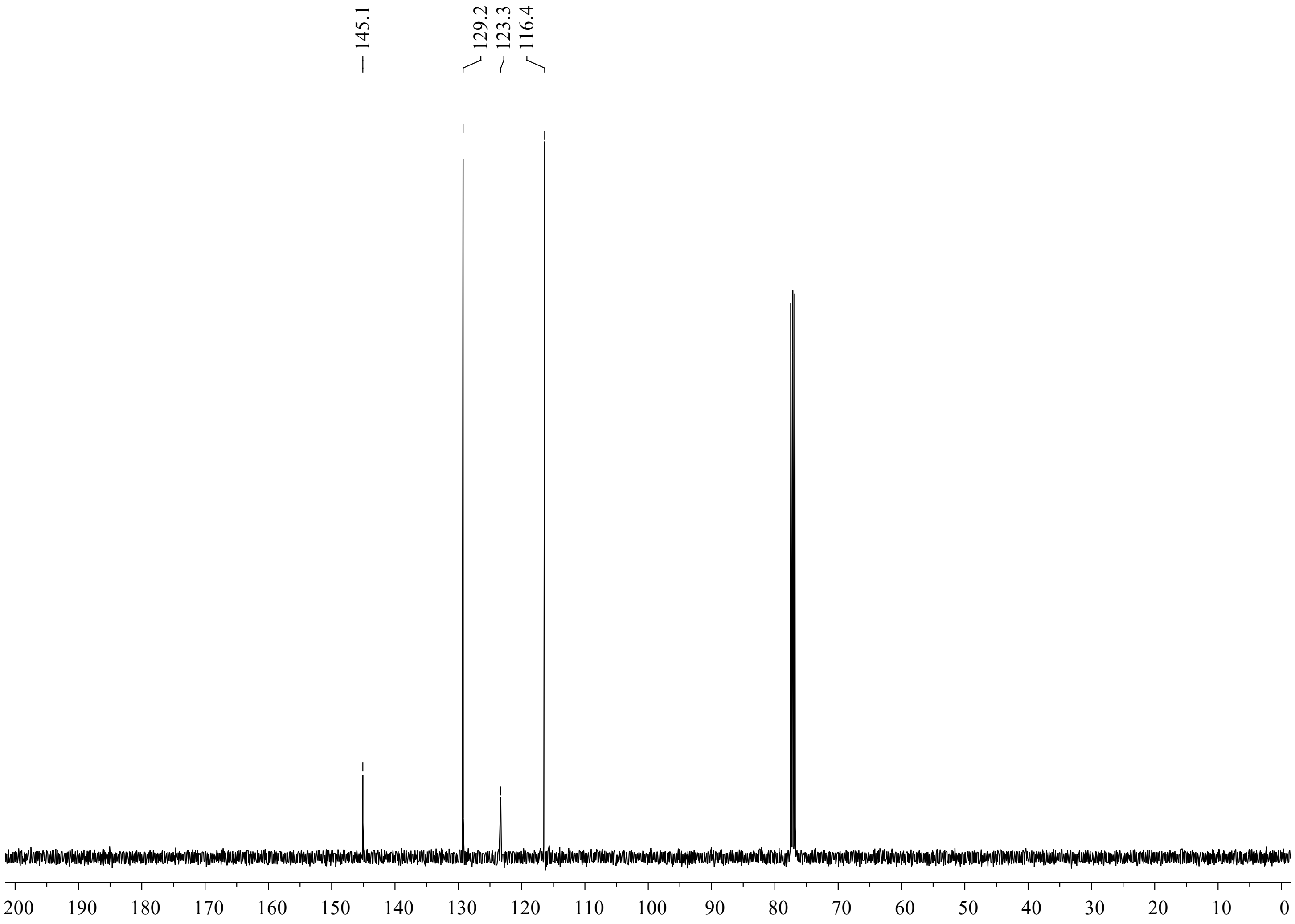

^{1}H NMR. 5-aminoquinoline (2e)

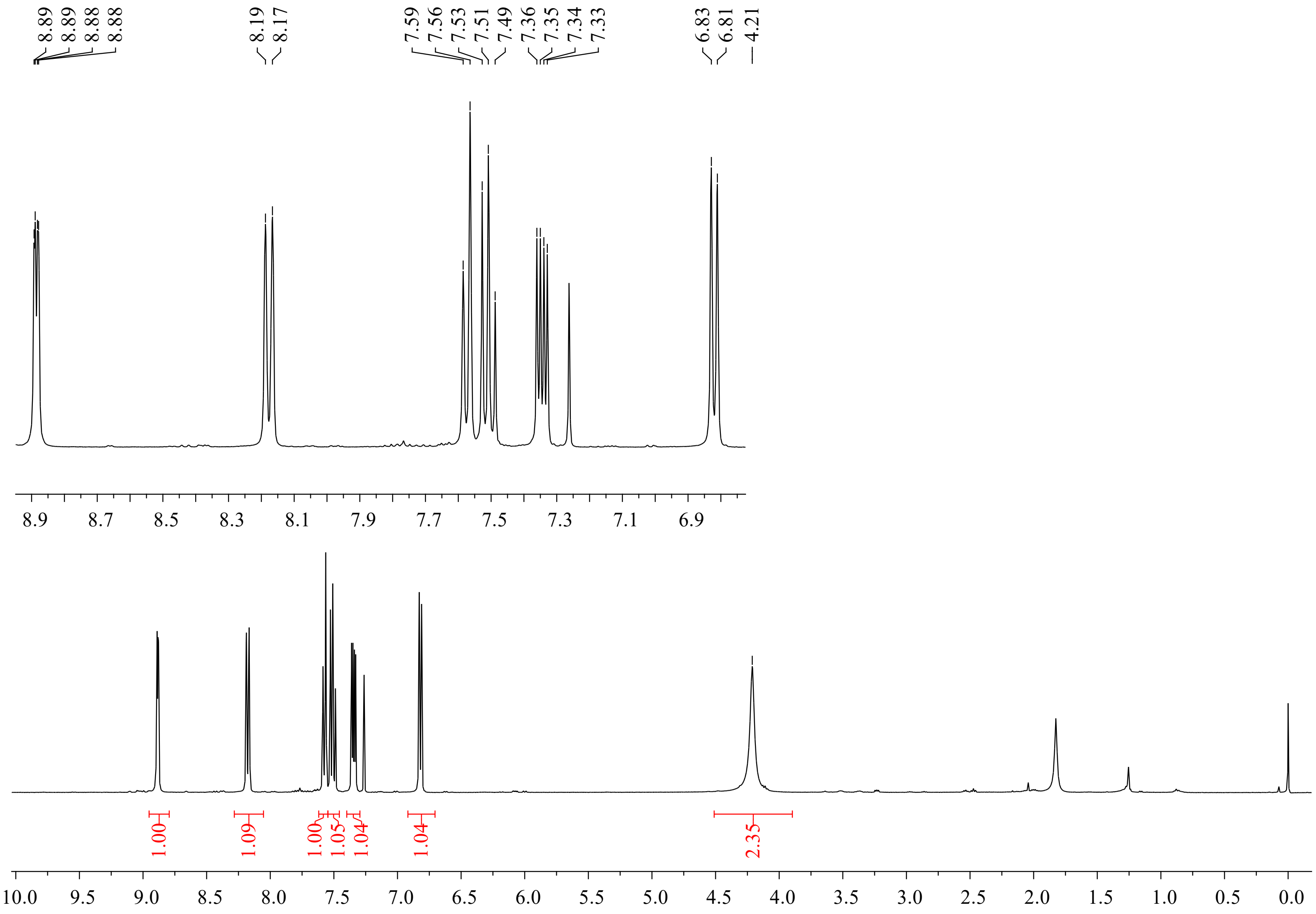

^{13}C NMR. 5-aminoquinoline (2e)

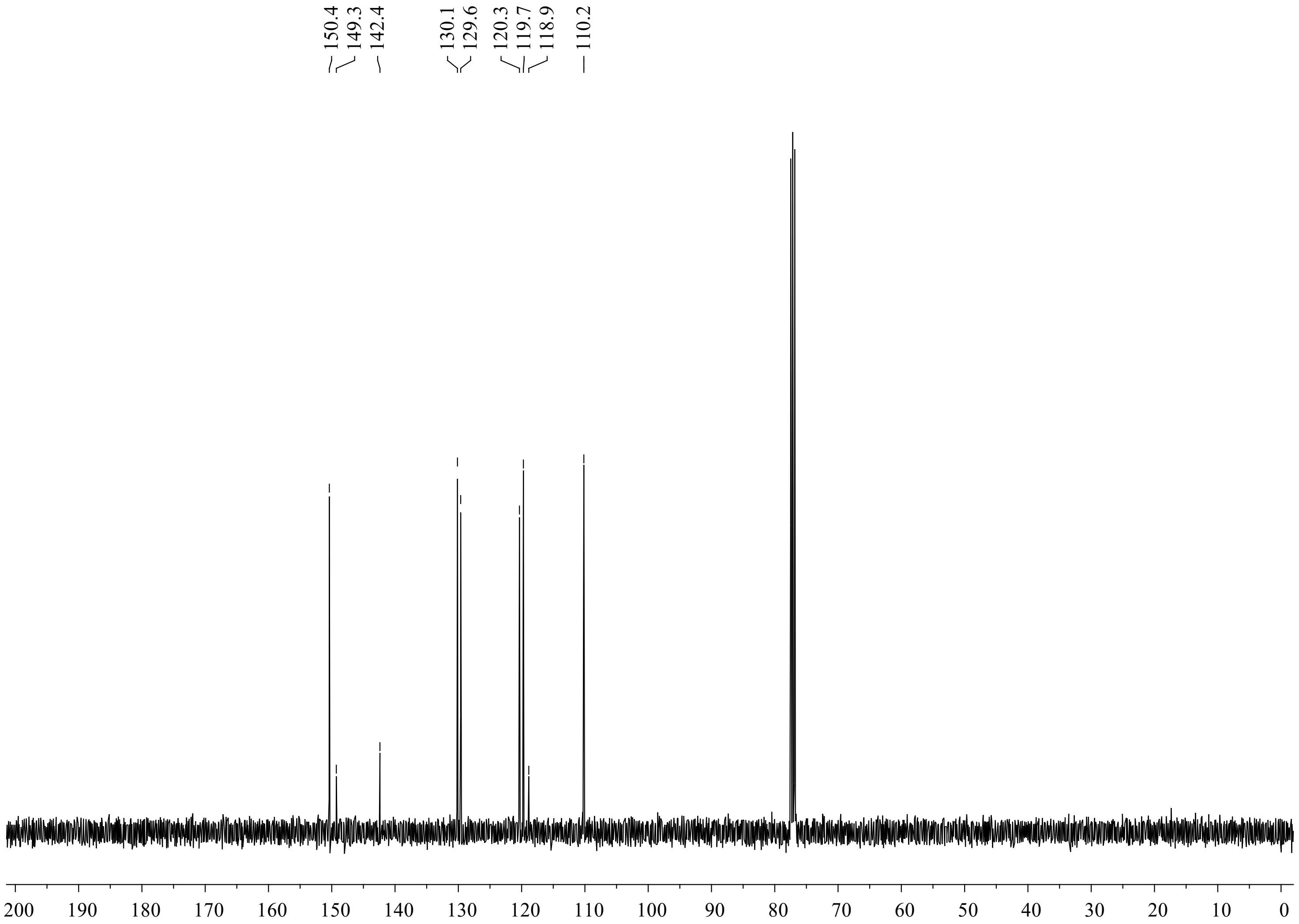

^{1}H NMR. 5-amino-1,2,3,4-tetrahydroquinoline (2e')

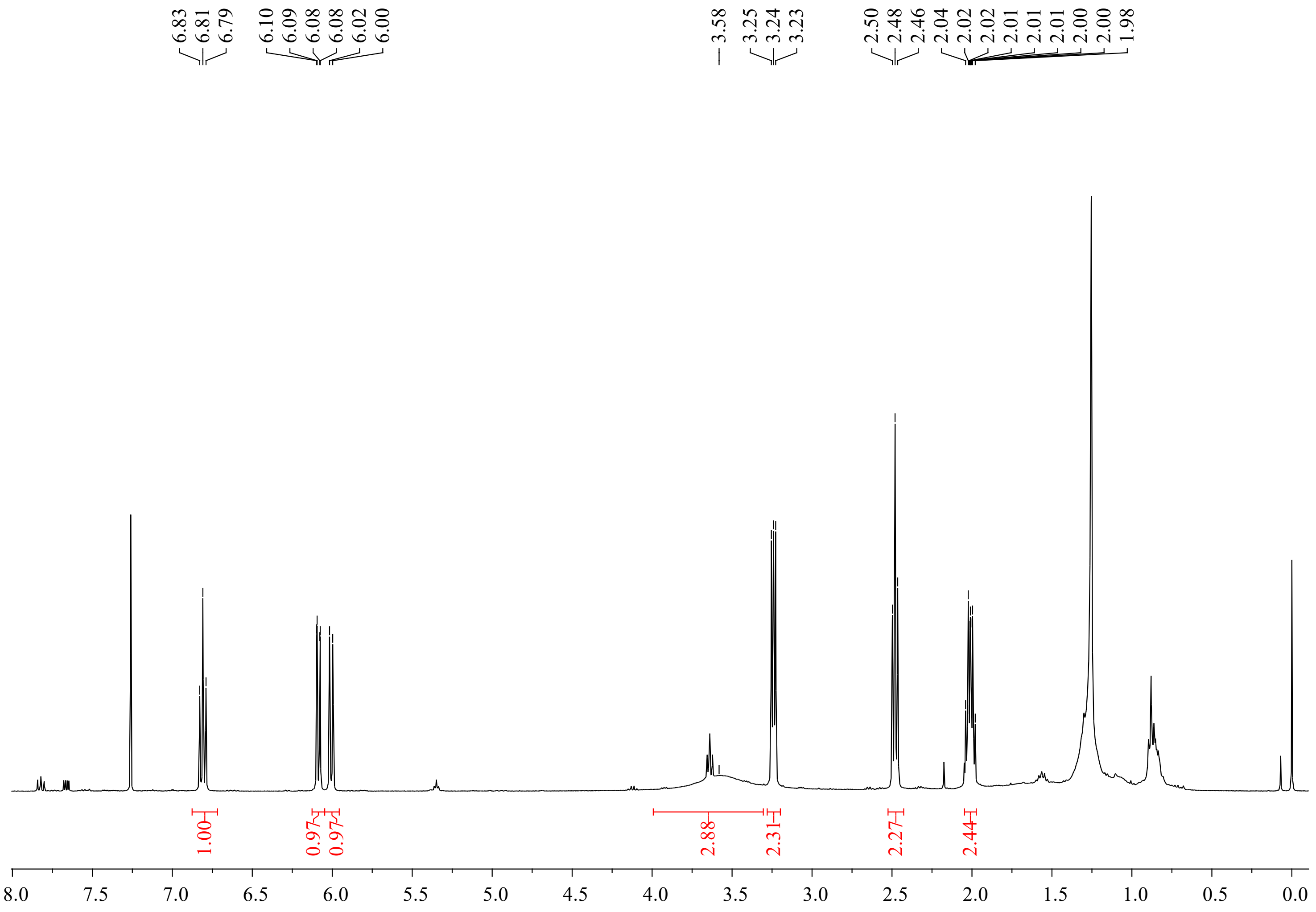

^{13}C NMR. 5-amino-1,2,3,4-tetrahydroquinoline (2e')

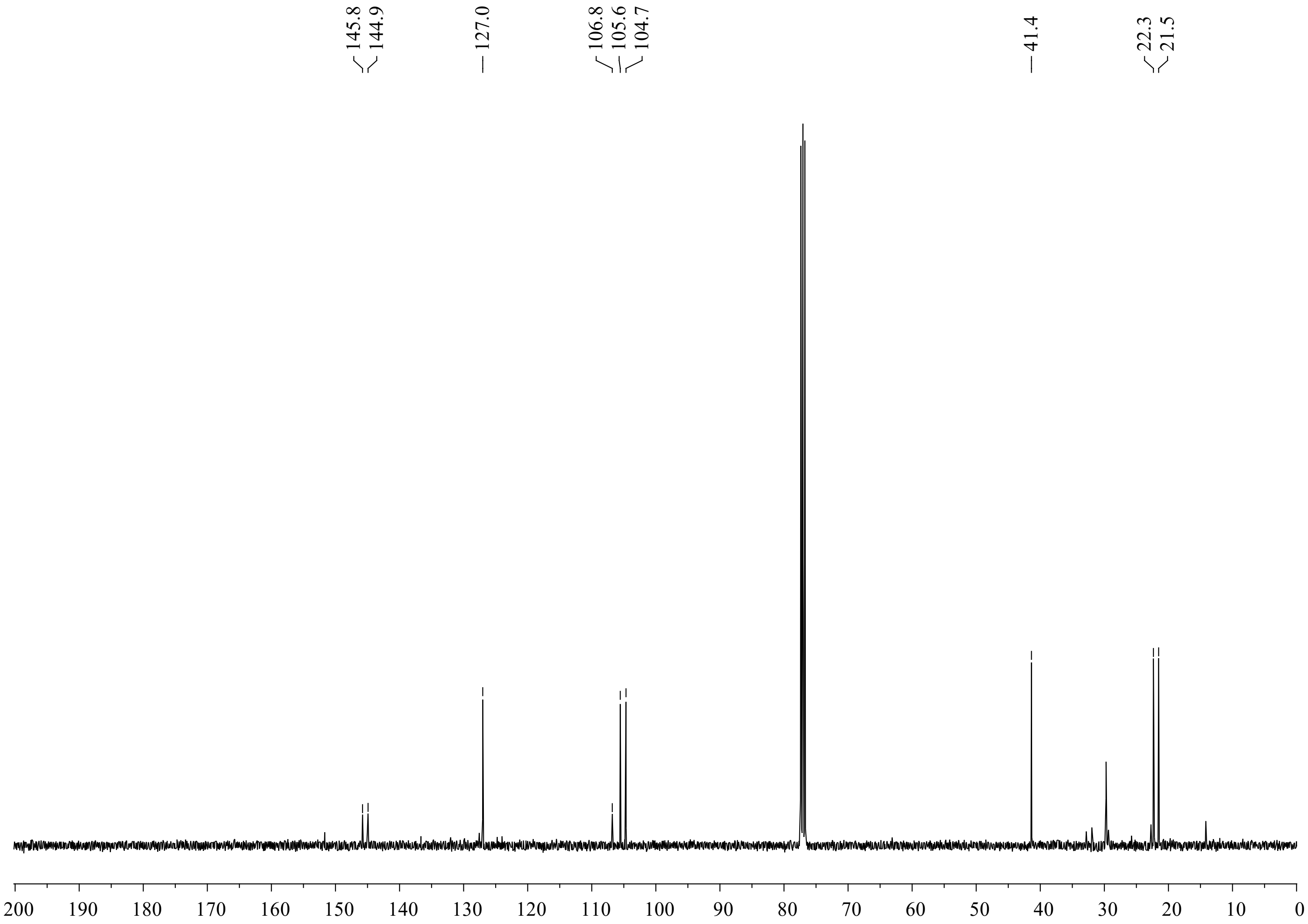

REFERENCES

1. Rehm, D.; Weller, A. Kinetics of Fluorescence Quenching by Electron and H-Atom Transfer. *Isr. J. Chem.* **1970**, *8*, 259-271.

2. Hari, D. P.; Konig, B. Synthetic Applications of Eosin Y in Photoredox Catalysis. *Chem. Commun.* **2014**, *50*, 6688-6699.

3. Chang, R. *General Chemistry*, 10th Edition; McGraw-Hill: New York, **2010**.

4. Murov, S. L.; Carmichael, I.; Hug, G. L. *Handbook of Photochemistry, Second Edition*; CRC Press: Hawthorne, CA, U.S.A., **1993**.

5. Romero, N. A.; Nicewicz, D. A. Organic Photoredox Catalysis. *Chem. Rev.* **2016**, *116*, 10075-10166.

6. (a) Mahata, A.; Rai, R. K.; Choudhuri, I.; Singh, S. K.; Pathak, B. Direct vs. indirect pathway for nitrobenzene reduction reaction on a Ni catalyst surface: a density functional study. *Phys. Chem. Chem. Phys.* **2014**, *16*, 26365-26374. (b) Liang, L.-Y.; Kung, Y.-H.; Hsiao, V. K. S.; Chu, C.-C. Reduction of Nitroaromatics by Gold Nanoparticles on Porous Silicon Fabricated Using Metal-Assisted Chemical Etching. *Nanomaterials* **2023**, *13*, 1805.
